# Constraining the chronology and ecology of Late Acheulean and Middle Palaeolithic occupations at the margins of the monsoon

**DOI:** 10.1038/s41598-021-98897-7

**Published:** 2021-10-05

**Authors:** James Blinkhorn, Hema Achyuthan, Julie Durcan, Patrick Roberts, Jana Ilgner

**Affiliations:** 1 Pan African Evolution Research Group, Max Planck Institute for the Science of Human History, Jena, Germany; 2 Department of Geography, Centre for Quaternary Research, Royal Holloway, University of London, London, UK; 3 Institute of Ocean Management, Anna University, Chennai, India; 4 School of Geography and the Environment, University of Oxford, Oxford, UK; 5 Department of Archaeology, Max Planck Institute for the Science of Human History, Jena, Germany

**Keywords:** Palaeoecology, Archaeology

## Abstract

South Asia hosts the world’s youngest Acheulean sites, with dated records typically restricted to sub-humid landscapes. The Thar Desert marks a major adaptive boundary between monsoonal Asia to the east and the Saharo-Arabian desert belt to the west, making it a key threshold to examine patterns of hominin ecological adaptation and its impacts on patterns of behaviour, demography and dispersal. Here, we investigate Palaeolithic occupations at the western margin of the South Asian monsoon at Singi Talav, undertaking new chronometric, sedimentological and palaeoecological studies of Acheulean and Middle Palaeolithic occupation horizons. We constrain occupations of the site between 248 and 65 thousand years ago. This presents the first direct palaeoecological evidence for landscapes occupied by South Asian Acheulean-producing populations, most notably in the main occupation horizon dating to 177 thousand years ago. Our results illustrate the potential role of the Thar Desert as an ecological, and demographic, frontier to Palaeolithic populations.

## Introduction

Acheulean technologies, characterised by the production of large bifacial tools, particularly handaxes and cleavers, are found across Africa and western Eurasia, first appearing ca. 1.75 million years ago (ma) in eastern Africa^1^ and persisting until ca. 130 thousand years ago (ka)^2^. During this timeframe significant changes are observed amongst hominin populations, such as geographic expansions^3–5^, demographic differentiation^6^, and encephalisation^7–9^. By the time Acheulean technologies were finally abandoned, substantial demographic complexity is observed amongst Eurasian hominin populations^10,11^. This includes expanding populations of *Homo sapiens*^12–14^ , Neanderthals in Europe and western/central Asia^4,15^, Denisovans in north/eastern Asia^16,17^, and late *Homo erectus* populations^18^ , the small-bodied *Homo floresiensis*^19^ and *Homo luzonensis*^20^ in South East Asia. The hominin demography of South Asia is poorly resolved until the appearance of *Homo sapiens* in the fossil record ca. 39–48 ka^21^, despite its central geographic placement in contrast to the range of other recent Eurasian hominin populations. Only a single fossil provides evidence for the earlier occupants of the region—best described as a Middle Pleistocene *Homo* population that shares mosaic features with populations to the east and west, which is associated with Acheulean technology^22,23^. In the absence of fossils, the transition in use of Acheulean to Middle Palaeolithic technologies provides crucial insights in to current debate surrounding the appearance of *Homo sapiens* in South Asia and their replacement of earlier hominin populations.

The antiquity of Acheulean habitation in South Asia is clearly demonstrated by archaeological evidence from Attirampakam, dating to 1.2–1.8 ma^24^, and Isampur, dating to > 1.2 ma^25^. The late persistence of the Acheulean in India and its replacement by Middle Palaeolithic technologies has only recently become a key focus of debate, however^26^. Acheulean technologies remain in use until ca. 212 ka in eastern Africa^27,28^ and ca. 190 ka in Arabia^29,30^, but the youngest Acheulean assemblages in the world are found at Patpara and Bamburi in the Middle Son Valley, India, dating between 140 and 120 ka^2^. Significant behavioural changes are observed across much of Africa and western Eurasia between 300 and 200 ka with the appearance of Middle Stone Age and Middle Palaeolithic assemblages^31–33^. Evidence across a range of sites and regions supports the appearance of Middle Palaeolithic technologies in South Asia considerably later, emerging from 114 ka onwards^34–38^, although recent studies from south-east India has been proposed as the earliest global evidence for Middle Palaeolithic technology ca. 385ka^39^. South Asia, therefore, presents a key global region in which substantial overlap between the use of Acheulean and Middle Palaeolithic technologies occurred demanding examination of patterns of technological innovation, ecological adaptation, and their relationships to demographic structure over this timeframe.

**Figure 1:**
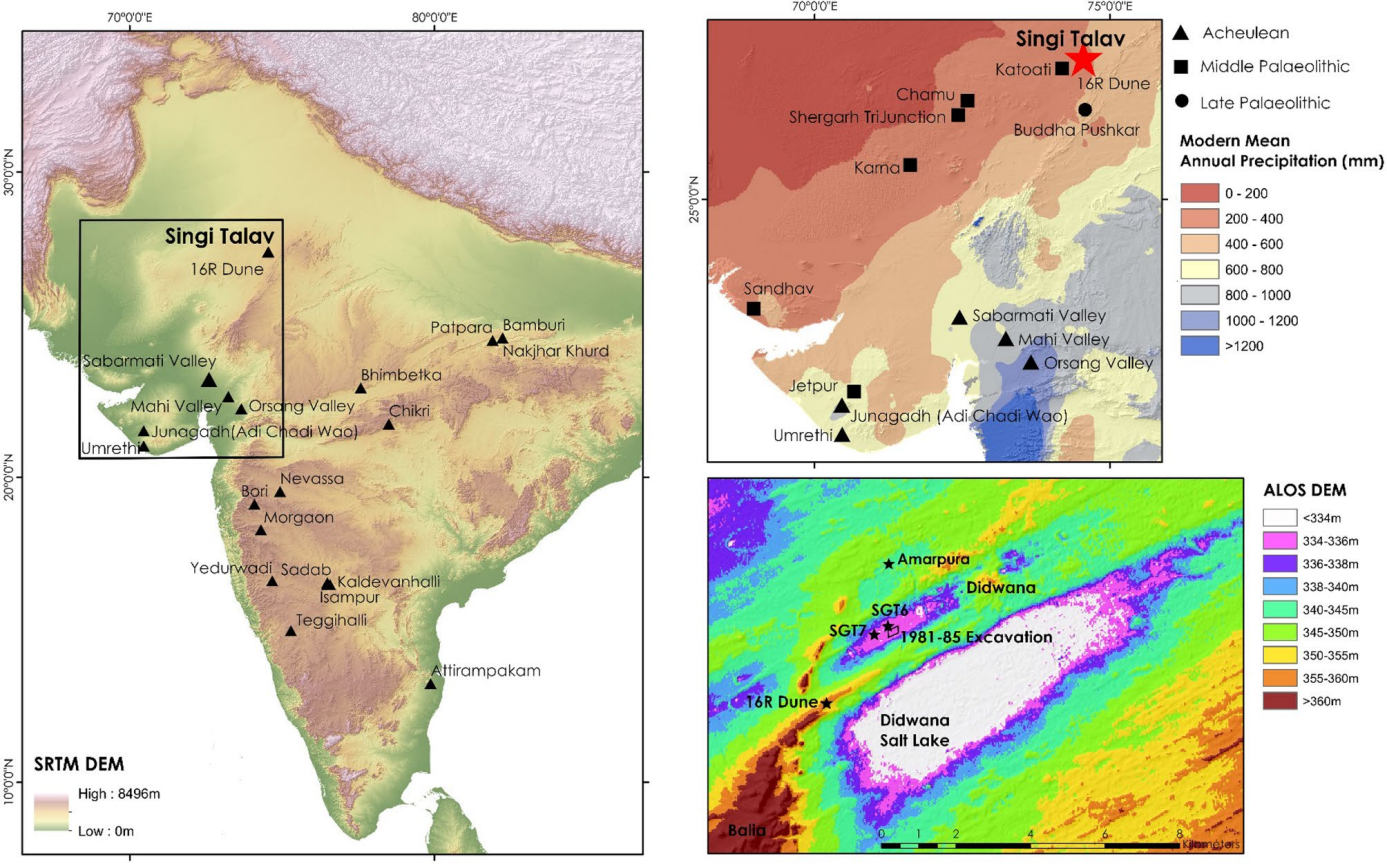
Maps highlighting the location of Singi Talav in relation to (left) key South Asian Acheulean sites [Data: SRTM (NASA)], (top right) and illustrating its position within semi-arid landscapes at the margin of the monsoon (Data: WorldClim.org^110^) with respect to other dated Palaeolithic sites in the Thar Desert region, (bottom right) with the location of SGT6 and SGT7 illustrated with respect to the 1981–85 excavation site and key landscape features [Data: ALOS (JAXA)] [Maps produced using ArcMap 10.5 (https://www.esri.com)]. Notably, the Singi Talav basin is separated from the adjacent Didwana salt lake basin by a linear dune formed within a break in the Balia hills; the excavation at 16R Dune exposes the depositional sequence of this dune, demonstrating ~ 18 m sand dune accumulation has occurred here beginning from ~ 187 ka.

Well-dated Acheulean sites remain sparse in South Asia, and are predominately located within the river basins of central and southern India (Fig. 1) where undated Acheulean sites are also abundant^40,41^. Palaeoenvironmental evidence from these sites is typically absent, yet central and southern India both receive considerable monsoonal rainfall that are likely to have supported sub-humid environments in spite of climatic flux throughout the Pleistocene. Western India marks a boundary of the modern monsoon system, with a sharp cline in precipitation present from the Aravalli range into the semi-arid to arid landscapes of the Thar Desert (Fig. 1)^42^. The presence of sand dunes well beyond the boundaries of the modern Thar Desert and into modern sub-humid zones signal the direct impact of palaeoclimatic flux on the region and its potential to disrupt habitability for Palaeolithic hominins^43^. Acheulean occupation, typified by the presence of handaxes and cleavers alongside sparser retouched flake tools, is restricted to the margins of western India, and is predominantly associated with larger, persistent rivers^44^. Meanwhile, Middle Palaeolithic occupation, characterised by the use of Levallois technology and retouched flake and point tool kits alongside sparse use of bifacial tools, is widespread across the region and includes dated sites present within the modern arid core of the Thar Desert (Fig. 1)^44^. As a result, the transition from Acheulean to Middle Palaeolithic appears to mark not only a change in the practices of stone tool production but also to the range of environments in which they were deployed. Examination of sites at the margins of the monsoonal zone is therefore critical for evaluating environmental adaptability amongst South Asia’s Acheulean populations.

Here, we present the results of a new study of the Palaeolithic occupations at Singi Talav (27°23′24.2"N 74°33′10.9"E; Fig. 1), undertaken in mitigation of immediate threats from industrial activity at the site. The lake-margin site was extensively excavated in the 1980’s, with archaeological assemblages from the upper 1 m of deposits described as microlithic (Layer 1), Middle Palaeolithic (Layer 2), and Acheulean (Layers 3–5) occupations, overlying a deeper sequence of lacustrine sediments (Layers 6–11) ^45–48^ (See SI1). The assemblages from Layers 3–5 are best described^49^, although the limited description of core technologies limits the potential to explore trajectories of change and innovation within these assemblages and the overlying Middle Palaeolithic (Layer 2) horizon. The sediment sequence at Singi Talav has not previously been directly dated, although a connection has been made to Amarpura Quarry^50,51^ where experimental Electron Spin Resonance dating returned a notional age estimate of 797ka^52^ from a carbonate rich sequence (See SI2). However, given experimental issues with the sample concerned^52^, the absence of any demonstrable stratigraphic correlation over the ca. 2 km between sites^53^, and substantial topographic differences between them (Figure SI1), no direct correlation is tenable. We present the results of new sedimentological and palaeoecological studies at Singi Talav and directly date the artefact bearing horizons using Optically Stimulated Luminescence. We explore the implication of these results for examining patterns of behavioural change in Palaeolithic South Asia and their relationship to wider trends in Eurasian hominin demography and palaeoecology.

## Results

### Sediment sequence

**Figure 2:**
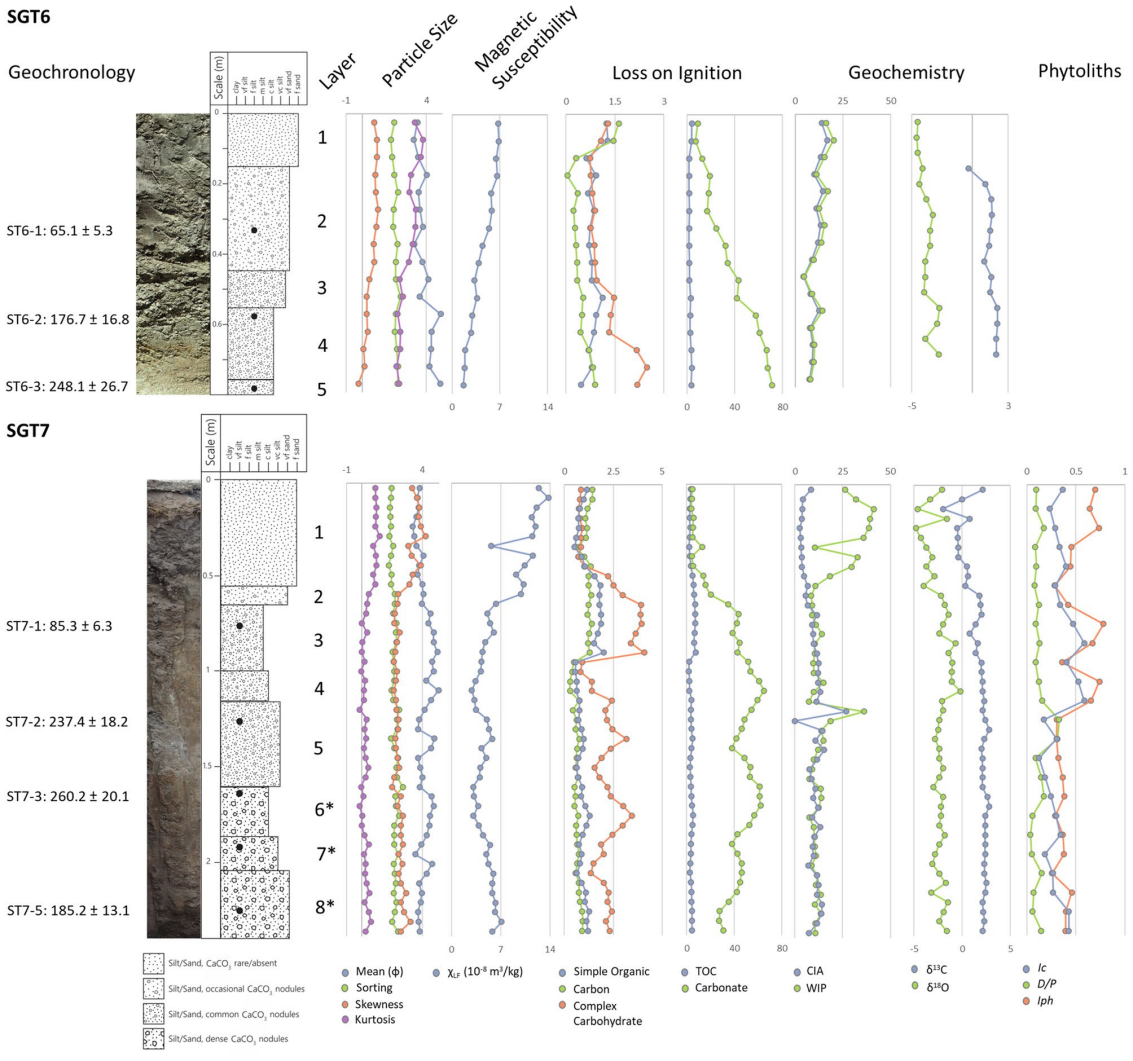
Sediment sequences exposed at Singi Talav [SGT6 (top) and SGT7 (bottom)], synthesising (from left to right) results of geochronology, section photo, sediment log (illustrating the location of dating samples), identification of layers, grain size summary statistics, magnetic susceptibility, loss on ignition results differentiating between alternate organic components, and between total organics (TOC) and carbonates, geochemical weathering indices (CIA; WIP), results of stable isotope analyses on carbonate nodules, and phytolith analysis (for SGT7 only). Layers 6*, 7* and 8* at SGT7 are broadly comparable to previous reports, but we use this notation in identification of some minor differences, whereas all other layers provide a direct match to previous reports. See SI3 and SI6 for further description and datasets.

We examined two newly exposed sediment sections at the Singi Talav locale (Fig. 2; see “Methods”), revealing a common stratigraphic sequence with previous investigations (see SI3). The first site, a 0.8 m deep section which we designated SGT6, is located in immediate proximity to previous excavations, where human and non-human activity have eroded the edges of previously excavated sections. Surface deposits at SGT6 comprise an upper aeolian sand containing sparse, soft, small carbonate nodules (Layer 1: 0–0.15 m) overlying a series of lacustrine deposits with distinct changes in frequency, size, induration and bridging of carbonate nodules present, alongside visible changes in sediment colour. The second deposit (Layer 2: 0.15–0.45 m) is a pale grey silty sand matrix preserving common, small, firm carbonate nodules, with a discrete artefact bearing horizon at 0.35 m, that is distinct from the underlying horizon (Layer 3: 0.45–0.55 m) based on the increased density, size and compaction of fine carbonate nodules, which also includes fresh lithic artefacts. Layer 4 (0.55–0.75 m) comprises a pale grey fine sand to silt horizon including more compact large carbonate nodules, with sparser artefacts found throughout, overlying Layer 5 (0.75–0.8 m), a pinkish grey sandy silt rich in powdery calcrete, fusing carbonate nodules and rarer fine ferric nodules, yielding a single lithic piece.

Stepwise changes in the frequency and form of carbonate nodules are apparent in the sediment section, and broadly match the average increase in carbonate presence in the fine sediment fraction, comprising 9.7% (Layer 1), 24.2% (Layer 2), 42.6% (Layer 3), 63.3% (Layer 4) and 71.5% (Layer 5). With the exception of Layer 1, a comparable stepwise increase in total organic components is observed ranging from 1.9% in Layer 2 to 3.5% in Layer 5. A linear relationship exists between magnetic susceptibility results and the combined presence of organic and carbonates within fine sediments, suggesting observed variability with depth relates to the size of mineral component of fine sediments, rather than a change in sediment source. Layers 1 and 2 show higher values for both Chemical Index of Alteration (CIA; mean = 14.66 and 11.6 respectively) and Weathering Index of Parker (WIP; mean = 17.21 and 13.17 respectively), with a sharp change to Layer 3 (CIA mean = 6.61; WIP mean = 7.13), with comparable values in Layers 4 and 5 (CIA mean = 9.57 and 7.49 respectively; WIP mean = 10.62 and 8.1 respectively). Layers 1–5 at SGT6 directly match descriptions of Layers 1–5 reported from previous research at the site, including characterisations of fine sediment grain size and proportional presence of CaCO_3_, enabling robust correlations between these sequences (see SI1 and SI3).

SGT7 is located 400 m west of the earlier excavation site, where a deeper sediment sequence was revealed to a maximum depth of 2.35 m, leading to the identification of eight discrete layers. While the uppermost five layers are more clearly comparable to the SGT6 sequence and previous excavations, the lower three layers show differing grain size characteristics, likely resulting from differential inputs from aeolian activity. At SGT7, we refer to Layers 1–5, providing a close match to SGT6 and previous excavations, and Layers 6*, 7*, and 8* in recognition that they are broadly comparable to these layers from previous excavations, but with some different features. Layer 1 (0–0.55 m) comprises an aeolian fine sand deposit, with very rare, fine carbonates, overlying Layer 2 (0.55–0.65 m) a reworked aeolian sand with more frequent carbonate nodules. A change in the size and frequency of carbonate nodules marks the transition to Layer 3 (0.65-1 m), and between Layers 4 (1–1.15 m) and 5 (1.15–1.6 m), alongside the concentrated presence of powdery calcretes in the latter. A sharp contact with Layer 6* (1.6–1.85 m) is identified, a mid-orangish brown silt with dense, firm and large, bridging carbonate nodules, overlying Layer 7* (1.85–2.05 m), and upward coarsening mid reddish-brown silt to sand. The lowest observed deposits (Layer 8*: 2.05–2.35 m) is an indurated dark reddish brown silty sand with indurated carbonates and fine ferric nodules. Stepped increases in the presence of calcium carbonate is observed between the top five layers, followed by high but fluctuating levels in the lower horizons. A more complex pattern is observed for total organic content, with distinct, high levels identified in Layer 3, alongside more muted peaks in Layer 5 and 6*. With the exception of Layer 1, CIA and WIP values show limited change throughout the sequence, and, as at SGT6, magnetic susceptibility results vary with respect to the mineral component of fine sediments, suggesting a single shared sediment source.

Both sites reflect key changes in the sediment sequence previously reported from the site. In particular, the transition to carbonate rich deposits starting with Layer 3 is clearly evident at both sites, reflected in both the macroscopic presence of carbonates, and changes in composition of fine sediments. The presence of dense, powdery calcretes in Layer 5 enables close comparisons between SGT6 and 7, as well as earlier excavations, constraining the key archaeological horizons at the site. Previous excavations highlighted discrete changes in sedimentation below Layer 5, and a comparable break is observed below Layer 5 at SGT7, though with some divergence with respect to the originally reported sequences, potentially representing lateral variability in broadly similar geomorphic contexts. Discrete, rather than continuous, changes in the sequences may suggest that sediment deposition at the site was pulsed, rather than continuous, and may reflect phases of enhanced monsoonal intensity, potentially complicated by the removal of unconsolidated fine sediment through aeolian erosion.

### Luminescence dating

Luminescence samples were taken from Layers 2 (0.34 m), 4 (0.57 m) and 5 (0.78 m) at SGT6, and Layers 3(0.77 m), 5 (1.26 m), 6* (1.63 m), 7* (1.91 m), and 8* (2.26 m) at SGT7. Ages were calculated from potassium feldspar signals and range between 65.14 ± 5.28 ka and 260.21 ± 20.14 ka, with key luminescence dating results presented in Table 1 and further detailed in Methods and SI.5. The three ages at SGT6 are stratigraphically consistent, and the measured luminescence signals have properties considered suitable for dating, which are within the saturation limit of the technique (see SI.5). We date Layer 2 to 65.14 ± 5.28 ka, Layer 4 to 176.67 ± 16.83 ka, and Layer 5 to 248.14 ± 26.75 ka.

**Table 1:** Luminescence dating summary. All calculations made prior to rounding. For further data and details relating to the dating, see Methods and SI.

Sample	Depth (m)	Equivalent dose (Gy)	Dose rate (Gy ka^−1^)					Age (ka)
			Alpha	Beta	Gamma	Cosmic	Total	
ST6-1	0.34	188.96 ± 7.42	0.13 ± 0.05	1.72 ± 0.50	0.82 ± 0.05	0.23 ± 0.02	2.90 ± 0.24	65.14 ± 5.28
ST6-2	0.57	406.16 ± 8.79	0.09 ± 0.03	1.41 ± 0.41	0.59 ± 0.04	0.21 ± 0.02	2.30 ± 0.22	176.67 ± 16.83
ST6-3	0.78	485.6 ± 12.26	0.09 ± 0.03	1.19 ± 0.34	0.49 ± 0.03	0.19 ± 0.02	1.96 ± 0.21	248.14 ± 26.75
ST7-1	0.77	285.65 ± 13.16	0.22 ± 0.08	1.90 ± 0.54	1.04 ± 0.07	0.19 ± 0.02	3.35 ± 0.25	85.27 ± 6.26
ST7-2	1.26	754.34 ± 26.14	0.18 ± 0.06	1.89 ± 0.54	0.93 ± 0.06	0.18 ± 0.02	3.18 ± 0.24	237.44 ± 18.16
ST7-3	1.63	823.83 ± 27.99	0.23 ± 0.08	1.77 ± 0.50	1.00 ± 0.07	0.17 ± 0.02	3.17 ± 0.25	260.21 ± 20.14
ST7-5	2.26	683.39 ± 19.77	0.21 ± 0.08	1.70 ± 0.49	1.18 ± 0.08	0.16 ± 0.02	3.69 ± 0.26	185.20 ± 13.05

At SGT7 ages vary between 85.27 ± 6.26 ka and 260.21 ± 20.14 ka and increase with depth for samples OSL-ST7-1 to -3. Like SGT6, the signals measured from samples from SGT7 meet luminescence screening criteria, although some individual signals appear to be in saturation (see SI.4). The very limited amount of material available for dating precluded the further testing of these samples or the use of alternative signals for verification (when tested, quartz signals were found to be in saturation). With regards to the apparent age inversion at the base of the sequence (sample OSL-ST7-5), we believe this is as a result of high levels of Thorium measured for this sample (double that of the overlying OSL-ST7-3 sample [Table SI.5]), potentially linked to inputs from the dense concentrations of carbonate nodules within the sediment matrix in combination with fluctuating groundwater levels. Given this anomaly, we suggest that OSL-ST7-5 is at present, not as reliable as the other luminescence ages at both sites, and note that if the Thorium concentration at OSL-ST7-5 matched that of OSL-ST7-3, the resulting age would resolve the age inversion within uncertainties (see SI.4). We note the broad parallel in ages between the two sites, with dates appearing in stratigraphic order for Layers 2–6* across the two sites, constraining the key archaeological horizons and latter stages of sedimentation at Singi Talav to between ca. 65 and at least 260 ka.

### Palaeoenvironmental studies

We examined the paleoecology of the late Middle Palaeolithic and early Late Pleistocene deposits at Singi Talav, using stable isotope analysis of carbonate nodules and recording of phytoliths to illuminate the nature of vegetation at the site and patterns of change through time (Fig. 2; Methods; SI5). Significant comparability can be observed in stable isotope values between SGT6 and SGT7, both of which exhibit highly restricted ranges. Layer 1 exhibits the lowest values of δ^13^C (average SGT6 = 0.3‰; SGT7 = 0.2‰), with step changes occurring between Layers 2 (average SGT6 = 1.2‰; SGT7 = 1.8‰) and 3 (average SGT6 = 1.2‰; SGT7 = 1.6‰) with Layers 4 (average SGT6 = 1.9‰; SGT7 = 2.1‰) and 5 (SGT6 = 2 ‰; average SGT7 = 2.2‰), and the highest values occurring in the lower deposits at SGT7 (Layer 6* mean = 2.5‰; Layer 7* mean = 2.4‰; Layer 8* mean = 2.3‰). These consistently high values are indicative of the dominance of C_4_ plant communities. A decrease in δ^18^O is observed through the SGT6 sequence from Layer 1 (mean = − 4.5‰) to Layer 5 (− 2.8‰), with a comparable pattern in Layer 1 (mean = − 3.4‰) to Layer 4 (mean = − 0.7‰), at SGT7, whereas in Layer 5–8*mean δ^18^O ranges from − 2.1‰ to − 2.7‰.

Phytolith assemblages were only studied from SGT7 (Table SI13), which show good preservation of diverse diagnostic types, with rarer occluded or patinated forms and poorer preservation of *Panicoid* phytoliths. Limited variability in the *D/P* index^54^ is observed throughout the sequence, with the exception of a clear peak in the upper deposits of Layer 5, suggesting an increase in woody vegetation, and lower levels observed associated with the lowest two units. Amongst C_4_ grasses, the *Iph* index^55^ indicates short forms comprise two thirds of the assemblage in Layers 5–8*, tall forms comprising 60–75% of the assemblage in Layers 4–3, short forms comprising 60% of the Layer 2 assemblage, and equal proportions appearing in Layer 1. The *Ic* index^56^ suggests grasses are dominated by C_4_ types throughout the sequence, ranging from 62 to 84% of the grass assemblage, and are most prevalent in Layers 3 and 4. In contrast, C_3_ grass types were most prevalent in Layers 5 and 8*.

The phytolith record thus illuminates greater variability in floral communities than the stable carbon isotope record. This may partly be explained by the seasonality of warm and dry conditions required for carbonate formation within a monsoonal climate offering a partial, seasonally selective record of vegetation that provides a C_4_ biased record^57^. Nevertheless, a clear focus on C_4_ vegetation is supported by both proxies throughout both sequences, highlighting that Palaeolithic occupations of the lake-edge occurred when the site was dominated by grasses capable of surviving the dry season to thrive in the warm and humid summer monsoon.

### Lithic artefacts

**Figure 3:**
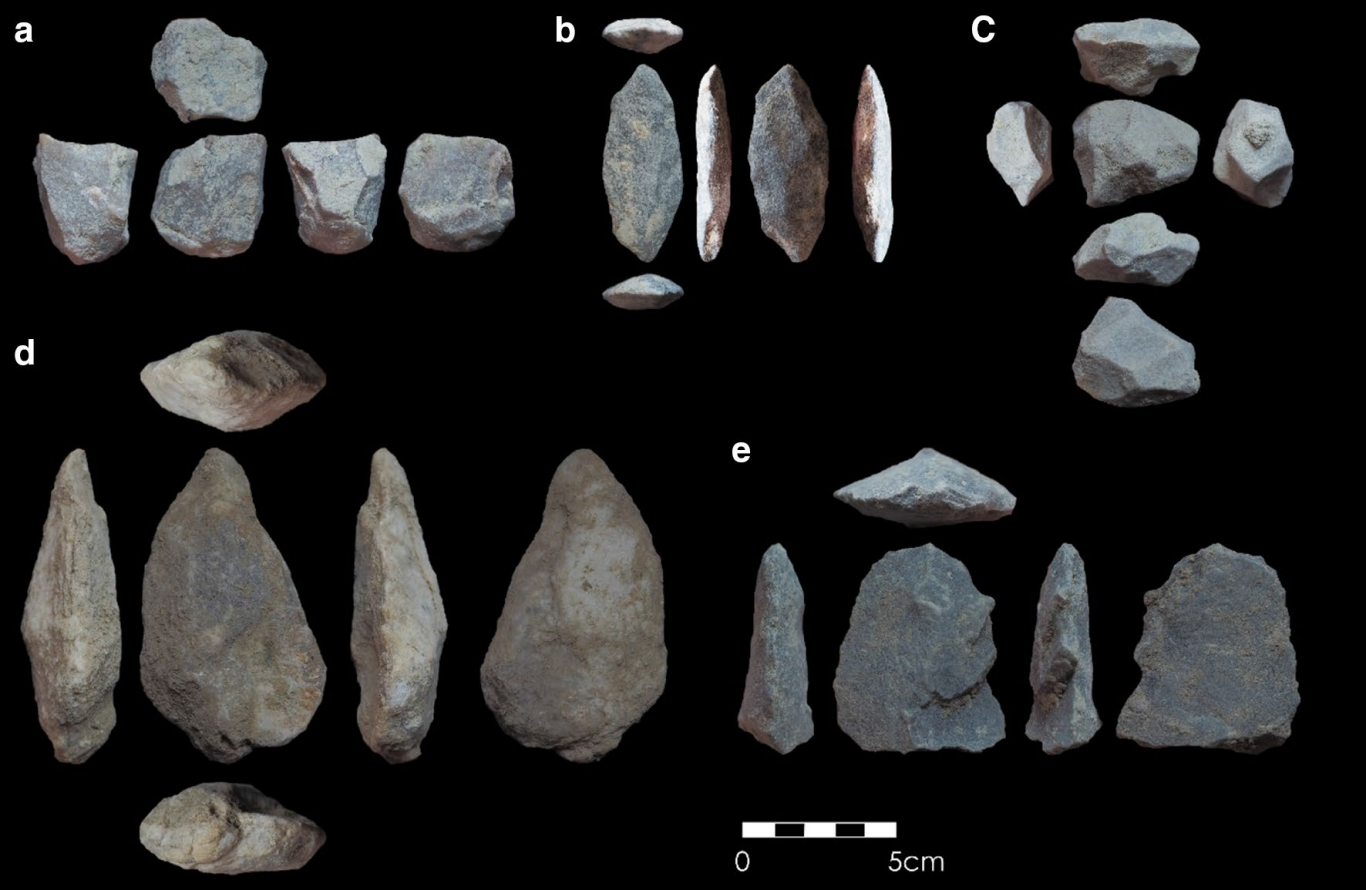
Artefacts recovered during new investigations at Singi Talav, including **(a)** multi-platform core (Layer 2); **(b)** bifacially retouched flake (Layer 3); **(c)** discoidal core (Layer 4); **(d)** biface with invasive removal (eroding from quarry edge); **(e)** finely retouched flake (eroding from quarry edge).

New collections of lithic artefacts were only encountered at SGT6 (Fig. 3). Weathered artefacts are identified at the contact between Layers 1 and 2, predominately comprised of small flaked pieces (n = 12) and broken flakes (n = 7), with a smaller number of simple cores (n = 5), focusing on cores on flakes. A discrete artefact horizon was identified between 0.3 and 0.35 m in Layer 2 comprised of larger complete flakes (n = 9), larger cores (n = 3) including multiplatform cores, one of which appears to be a weathered and broken centripetal preferential Levallois core, with sparser flaked pieces (n = 4). A smaller artefact collection was recovered from the thinner deposits of Layer 3, including a large multiplatform core (length = 90 cm) and a large simply retouched flaked piece (length = 68 cm) alongside four other pieces. The artefact collection from Layer 4 includes five flaked pieces, two broken flakes and one complete flake, and a single discoidal core. A single complete flake was recovered from Layer 5. These finds are consistent with artefact assemblages recovered from the substantially larger, earlier excavations, though diagnostic technological elements are limited to the Layer 2 broken Levallois core, dating to 65.14 ± 5.28 ka. The Layer 3 assemblage is not directly dated at SGT6 but corresponds to the age of 85.27 ± 6.26 ka from SGT7. The Layer 4 assemblage is directly dated to 176.67 ± 16.83 ka, with the single artefact from Layer 5 dating to 248.14 ± 26.75 ka and closely matches the corresponding age of 237.44 ± 18.16 ka from SGT7. The main archaeological sequence at Singi Talav overlies the date of Layer 6* from SGT7 of 260.21 ± 20.14 ka.

## Discussion

Our results place Palaeolithic occupations at Singi Talav within a clear chronometric and ecological framework for the first time. Critically, our new luminescence ages directly date occupations at the site to ~ 248 ka, ~ 177 ka, ~ 85 ka and ~ 65 ka. The oldest major occupation identified at Singi Talav (Layer 5) significantly pre-dates comparable evidence from elsewhere in the Thar Desert region^44^, and spans a timeframe in which no directly dated sites are known across South Asia, but is younger than dated Acheulean occupations at Sadab (ca.290 ka) and Teggihalli (ca. 287 ka)^58^, Nevassa (> 350 ka)^59^ and Yedurwadi (> 350 ka)^60^, as well as the oldest Middle Palaeolithic occupation reported at Attirampakam^39^. More intensive occupation of Singi Talav is attested to by the larger lithic assemblages from Layer 4, dating to early MIS 6 (~ 177 ka). This is contemporaneous to the presence of hominin activity at 16R Dune^61^, and matched by evidence for Acheulean activity elsewhere in the Thar Desert (e.g. Junagadh [Adi Chadi Wao] & Umrethi^62^; the Mahi, Orsang and Sabarmati Valleys^63–65^) and across South Asia (e.g. Patpara and Bamburi^2^; Bhimbetka^66^; Kaldevanhalli-I^58^) at the end of the Middle Pleistocene, alongside the reported presence of Middle Palaeolithic technology at Attirampakam^39^. Layers 3 and 2 mark early Late Pleistocene occupation of the site, dating to ~ 85 ka and ~ 65 ka respectively, which are broadly coincident with Middle Palaeolithic occupations of the nearby sites of 16R Dune (80–40 ka^61^) and Katoati (96–45 ka^35,67^), as well as a range of Middle Palaeolithic sites across the Thar Desert and South Asia^26^. Dating of the Palaeolithic occupations at Singi Talav, therefore, demonstrates the antiquity of inhabitation of western India and spans a critical timeframe for examining the Acheulean to Middle Palaeolithic transition across the subcontinent.

Layers 5–3 at Singi Talav have been attributed to the Acheulean (SI4), in part reflecting the acceptance of an Early Pleistocene chronology^68^. The limited available description of the Layer 5 assemblage, its relatively small size, the prevalence of flaking debris, and scarcity of larger tools, are not directly diagnostic of Acheulean technology, but are consistent with such an attribution given their chrono-stratigraphic context. The Layer 4 assemblage contains a high proportion of handaxes (n = 18) and includes cleavers (n = 3) and other large cutting tools, with further evidence for a focus on bifacial reduction evident in the debitage assemblage^49^, which are features consistent with contemporaneous Acheulean assemblages from across South Asia. Singi Talav Layer 4 clearly documents the late persistence of Acheulean technology in South Asia, here dating to 177 ka, post-dating the most recent Acheulean occupations of either Arabia^30^ and eastern Africa^27^, making it one of the youngest Acheulean sites worldwide. The assemblage is also notable for the presence of a collection of six quartz crystals intentionally transported to the site with no clear utilitarian purpose^69^ and therefore mark the oldest dated non-utilitarian objects in the South Asian record at present. The Layer 3 assemblage includes a small number of handaxes (n = 3), but lacks other clear features that are directly diagnostic of Acheulean technology (e.g. the presence of cleavers), alongside a greater focus on core technology. An Acheulean occupation at Singi Talav at 85 ka is consistent with recent models suggesting late continuity of Acheulean and substantial overlaps with the Middle Palaeolithic across Asia^70^. However, the sparse use of bifacial tools alongside greater focus on diverse core reduction technologies is now shown to be a consistent feature of the Thar Desert Middle Palaeolithic^44^, such as at 16R Dune 80–40 ka^61^, with the earliest Middle Palaeolithic occupations in the region appearing from 114 ka^34^. Further direct study of this assemblage is required to adequately resolve whether it presents a consistent character with other young Late Acheulean or older Middle Palaeolithic assemblages, or a mosaic of features indicating more complexity than presently documented for this cultural transition. This ambiguity prohibits assigning the Layer 3 assemblage as the youngest Acheulean in South Asia or the world.

**Figure 4:**
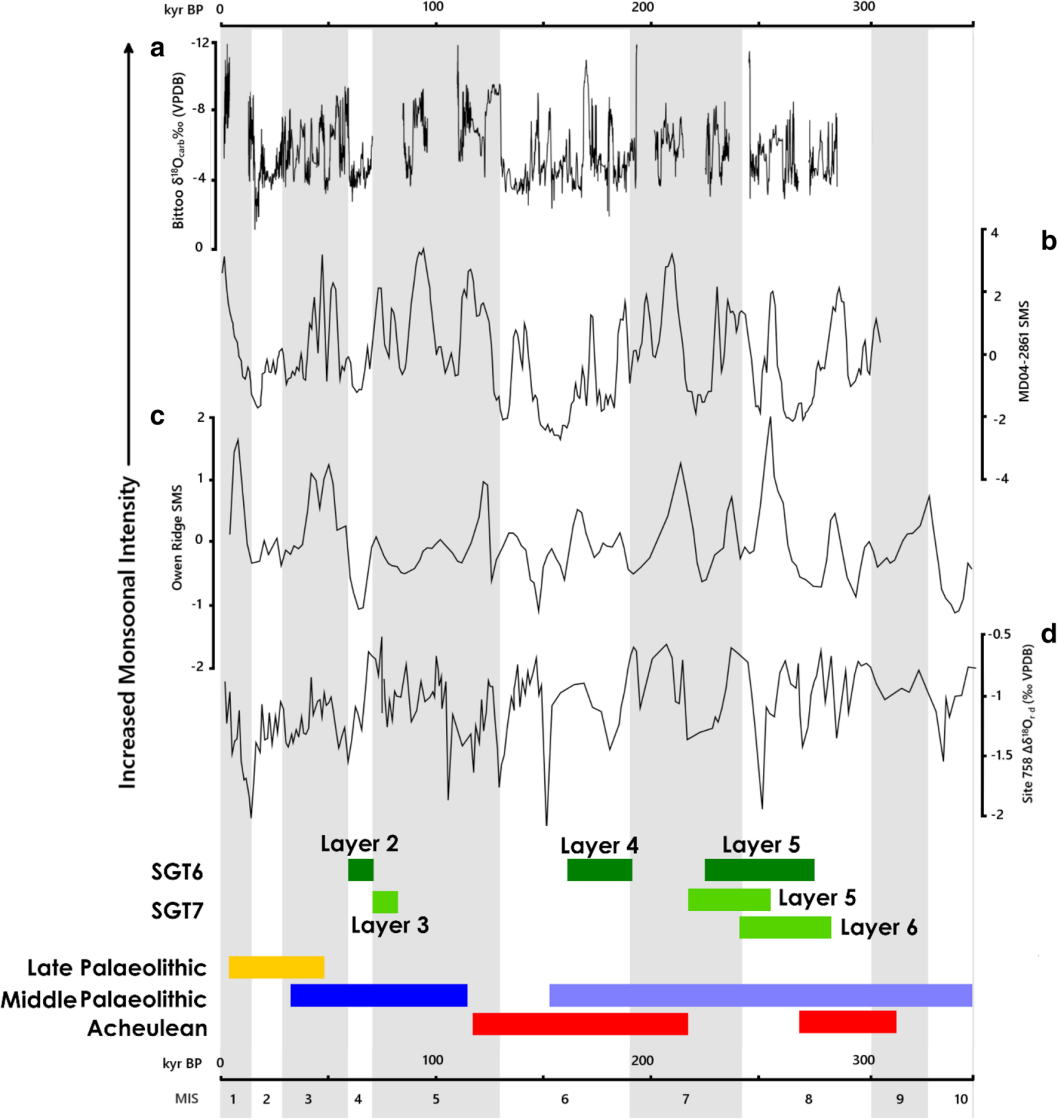
High altitude cave speleotherm (**a**: Bittoo Cave [India]^110^) and marine core records from the Arabian Sea (**b**: MD04^111^; **c**: Owen Ridge^112^) and Bay of Bengal (**d**: Site 758^113^) illustrating patterns of monsoonal variability spanning the occupations of Singi Talav (SGT6: dark green; SGT7: light green) and major phases of cultural activity in South Asia including Late Palaeolithic (yellow) and Middle Palaeolithic (dark blue)^26^, Attirampakam Middle Palaeolithic (light blue [NB only two assemblages present])^39^, and Late Acheulean (red)^2,58,62^. Phases of lacustrine deposition at Singi Talav are associated with both prominent peaks and troughs of monsoonal intensity during glacial phases (MIS 8, 6 and 4) evident in Arabian Sea records, highlighting the importance of terrestrial proxy records to understand how wider patterns of climatic flux may have been manifest in landscapes occupied by Palaeolithic hominins.

Our research presents the first detailed study of lacustrine sedimentation in South Asia that extends into the Middle Pleistocene, including the first comparison of alternate palaeoecological proxies to contextualise Middle Pleistocene archaeological sites. Palaeoecological evidence for humid phases in the Thar Desert region has been largely restricted to Holocene and terminal Pleistocene lake records^71–77^, with broader palaeoenvironmental evidence available from Late Pleistocene fluvial sequences^34,63,78–80^. More limited evidence for Middle Pleistocene environments derive from studies of aeolian^78,81,82^ and fluvial^83^ activity, the chronology of which is exceeded by this study of Singi Talav. MIS 6 occupations at Singi Talav are, however, contemporaneous with evidence for fluvial activity in the central Thar Desert^83^. The presence of active lakes and rivers may have presented a substantially different structure of regional ecology and habitability for hominins in contrast to the modern constellation of playas and ephemeral or seasonal streams. The accretion of the upper 18 m of SW-NE linear dune over the past 187 ± 43 ka, exposed at 16R Dune^81,84^, demonstrates the potential for aeolian activity to disrupt such humid landscape features during the course of occupation of the site, and potentially separated Singi Talav from the larger, adjacent, Didwana lake during the Middle Pleistocene (Fig. 1c). Yet the phytolith and stable isotope records demonstrate that phases of lacustrine deposition at Singi Talav supported C_4_ floral communities, which thrive under the seasonally hot, humid conditions promoted by a strong, summer monsoon regime. Wider evidence from across the Thar Desert supports a pattern of Palaeolithic occupation associated with comparable flora and associated with peaks in monsoonal intensity^34,85^. The accumulation of lake deposits and flourishing C_4_ ecologies at Singi Talav spans both peaks and troughs in monsoonal intensity, including episodes of dissonance between Arabian Sea and Bay of Bengal records (Fig. 4). This highlights the importance of terrestrial records to resolve the environmental context of hominin occupations, and particularly in landscape characterised by significant flux, such as the monsoonal threshold in western India. Our study provides the first direct evidence from South Asia to demonstrate Acheulean populations directly engaged with landscapes at the margins of the monsoon.

**Figure 5:**
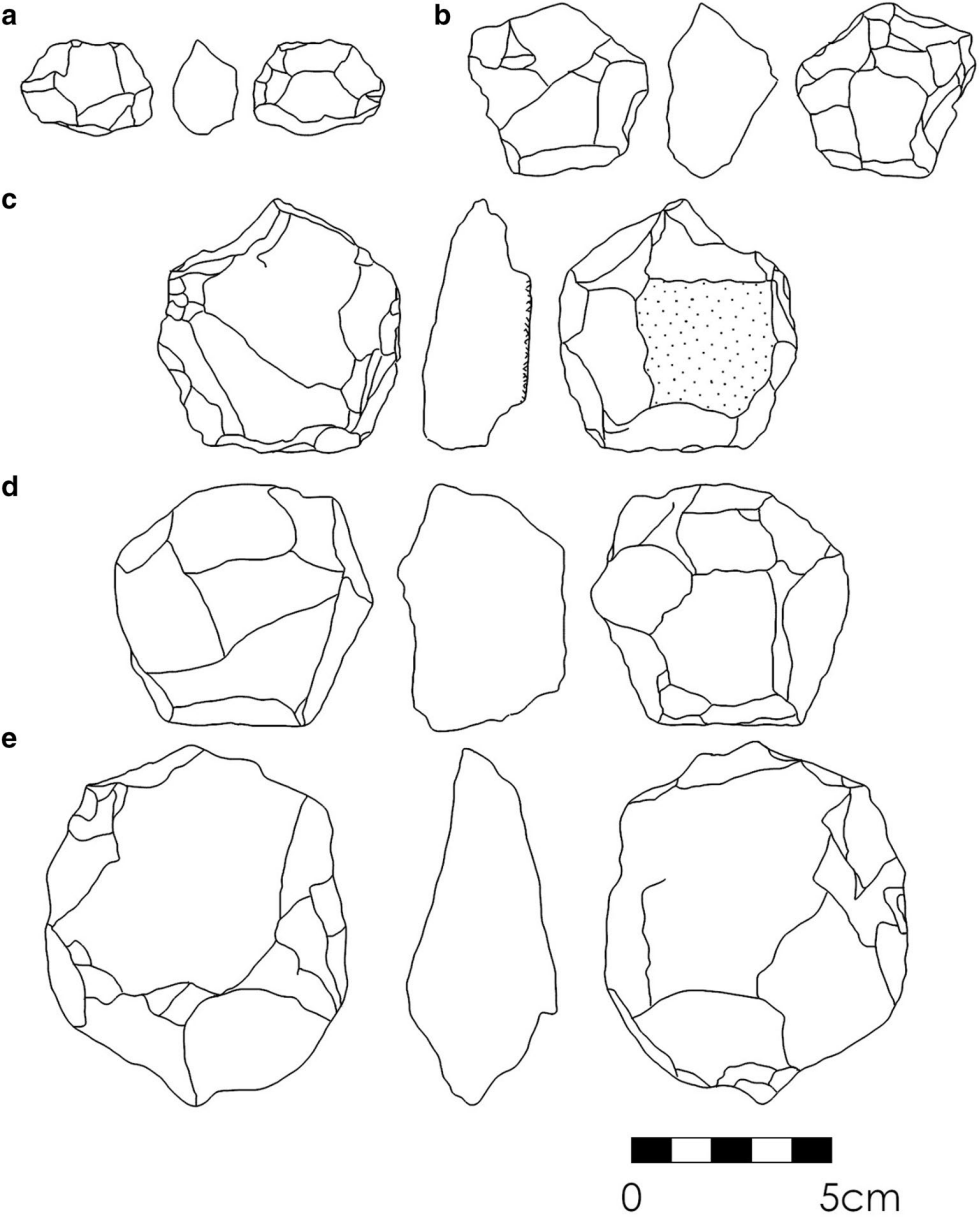
Hierarchical bifacial cores reported from Singi Talav assemblages Layer 3 (**a**) and Layer 4 (**b**–**e**) (redrawn from ^49^).

Comparative studies of bifaces at multiple South Asian Acheulean sites have suggested a trend of increasing refinement (defined as the ratio of thickness to width) through time^86^. However, poorly refined bifaces from the final Middle Pleistocene occupations at Singi Talav confound this pattern. Preferential and invasive flaking of bifaces and bifacial cores, including piecemeal evidence for Levallois technology, is highlighted amongst the youngest Acheulean sites in South Asia^86,87^. Comparable artefacts appear to occur at Singi Talav (Figs. 3d, 5), further supporting recognition of one of the youngest Acheulean assemblages in the world. This may be indicative of broad changes in technological practice shared amongst late Acheulean populations, adaptations to the unique ecological challenges at the margins of the monsoon at Singi Talav, or a combination of both. The occurrence of young Acheulean occupations in west and central India contrasts with the recent report of an early Middle Palaeolithic in south-east India, dating between ca. 385–172 ka^39^, suggesting an overlap of ca. 255 thousand years. In eastern Africa, an overlap of ca. 90 thousand years is observed between the youngest Acheulean (ca. 212 ka^27,28^) and earliest Middle Stone Age (ca. 300 ka^31,88^) sites, with the piecemeal occurrence of Levallois technology in Acheulean assemblages appearing from 600 to 500 ka^88–90^. A comparable ca. 60 ka overlap between the youngest Acheulean (ca. 190 ka^29,30^) and the oldest Middle Palaeolithic (ca. 250 ka^32^) is also observed in South West Asia, where no such continuity in the use of Levallois technology is witnessed. Eastern Africa and South West Asia present productive alternate models for the Acheulean-Middle Palaeolithic transition for comparisons to South Asia, with the former likely representing autochthonous changes and the latter argued to have resulted from population overturn^91^. Notably, in both instances, young Acheulean sites are found in more marginal, semi-arid habitats, potentially highlighting ecological structure to the Acheulean to Middle Palaeolithic transition. Further well-dated and excavated late Middle Pleistocene sites are required in order to robustly resolve between either alternative in the South Asian context, with this study from Singi Talav also demonstrating the potential importance of resolving the palaeoecological context of major cultural changes in this region.

The persistence of the Acheulean until the very end of the Middle Pleistocene in South Asia is remarkable and coincides with significant demographic and behavioural upheaval across Eurasia. The longevity of the Acheulean in South Asia is paralleled by the persistence of *Homo erectus* in Southeast Asia, the hominin typically associated with the initial spread of Acheulean technologies into Asia^40^. Genetic records for Denisovan populations also point to enduring patterns of demographic structure, with introgression from discrete Denisovan populations evident in modern populations suggesting distinct geographic distributions^92,93^. Modern South Asians preserve significant evidence for Denisovan introgression, in contrast to South West Asians^94^, raising the prospect that a Denisovan population inhabited South Asia during the timeframe of modern human expansions across Asia. Recent discoveries that suggest further demographic variability amongst late Middle Pleistocene Asian hominins complicate these issues further^10^. Regardless of which hominin populations produced Acheulean toolkits at Singi Talav at the end of the Middle Pleistocene, they had begun to engage with more marginal environments at the western edge of monsoonal Asia and the eastern edge of the Saharo-Arabian desert belt, a major biogeographic threshold^95^. Such ecological tenacity parallels records from South West Asia and eastern Africa, where the youngest Acheulean sites are found in the arid interior of Arabia or higher-altitude locations in the Ethiopian rift, at times when Middle Palaeolithic/Stone Age sites are found in less challenging settings, reflecting the enduring utility of Acheulean toolkits and adaptability of the hominin populations that used them. At Singi Talav this tenacity led to late survival of Acheulean technology, contemporaneous to the expansions of *Homo sapiens* into South West Asia and immediately prior to their eastward dispersals across Asia. The biogeographic threshold between the Saharo-Arabian desert belt and monsoonal Asia encountered by eastward expanding populations would have been further accentuated by a stark behavioural, and potentially demographic, frontier at the onset of the Late Pleistocene, that may have hosted a range of interactions between distinct hominin groups.

## Methods

### Fieldwork

Fieldwork was conducted in June 2016, with the intervention at SGT6 undertaken within a 1 × 1 m square using hand tools, distinguishing between discrete sediment units and subdividing units into 10 cm levels where necessary to ensure controlled recovery of any artefacts, supported by sieving of excavated sediments through 5 mm mesh. Digging below 0.8 m was prohibited by the appearance of groundwater, relating to use of the old quarry as part of industrial activity at the site. The sediment sequence was sampled at 5 cm resolution for diverse sedimentological and palaeoenvironmental studies. Opaque metal tubes were hammered into the section wall to recover samples for luminescence dating. Following sampling, the intervention was backfilled. At SGT7, a mechanical digger was removing modern silt, relating to industrial activity, and was able to reveal a fresh section of the sediment deposits to the base of the modern sump/silt trap. This was monitored for the appearance of archaeological material, which was absent. The sediment sequence was sampled at 5 cm resolution for diverse sedimentological and palaeoenvironmental studies. Opaque metal tubes were again hammered into the section wall to recover samples for luminescence dating.

*LPSA*: Individual sediment samples (~ 10 g) were sieved to remove particles larger than 2 mm and bathed in 1% HCl for a minimum of 24 h in a water bath at 90 °C to fully evolve carbonates and disaggregate fine material. Purified water was added to the samples, which were then centrifuged at 3500 rpm for 13 min, with the excess liquid decanted off. Samples were agitated on a whirlimixer and subsampled for measurement using a Malvern Mastersizer 2000, selected from the centre of the resultant vortex. Summary statistics and sediment descriptions were produced using Gradistat^96^.

### Loss on ignition

Fine sediment samples (~ 10 g; < 2 mm) were weighed and heated in a muffle furnace to 105 °C, 400 °C, 480 °C, 550 °C and 950 °C for 6 h, allowing sediments to cool to 105 °C for weighing at each interval and calculate the proportion of material lost from the dried (105 °C) sample at each interval relating to simple carbohydrates (400 °C), complex carbohydrates (480 °C), carbon (550 °C) and carbonate (950 °C) (following^97–99^). The remaining sample constitutes the total mineral residue, with total organic carbon comprising all material lost between 105 and 550 °C^100^.

### Constrained hierarchical clustering

The mean, sorting, skew and kurtosis of fine sediment fractions and the proportions of organic and carbonate fractions resulting from Loss on Ignition (see SI6 for data) were analysed using *chclust* function of *rioja* package ^101^ in *R*^102^, as an aid to resolve patterns of stratigraphic continuity. These results, combined with field observations on sediment colour, texture and composition (including macroscopic features such as size and density of carbonate nodules), were used to resolve between sediment units, correlate stratigraphic sections between SGT6 and SGT7, and identify corresponding layers from earlier excavations.

### Magnetic susceptibility

Magnetic susceptibility was measured in the laboratory using a Bartlington MS3 magnetic susceptibility meter coupled with the MS3B sensor to analyse 10 cm^3^ samples in plastic pots, weighed on precision scales to enable calculation of mass specific values following drying at 105 °C. Both high (4.6 kHz; HF) and low (0.46 kHz; LF) frequency magnetic susceptibility was recorded, enabling calculation of the percentage frequency dependant susceptibility (FD%). We identified a linear relationship between LF magnetic susceptibility values and the proportion of mineral residue from each sample, indicating that changing values in magnetic susceptibility values correspond to the non-mineral component of sediment samples, rather than a change in sediment source.

### ICP-OES

One cubic centimetre sediment samples were fully evolved in aqua regia in water baths at 90 °C.Sub-samples of the resulting supernatant were then analysed using a Perkin-Elmer ICP-OES, undertaking three replicate measurements and producing a mean concentration in ppm (mg/l) and relative standard deviation (%RSD).The Weathering Index of Parker (WIP^103^) was calculated using molar weights of each element as oxides according to the formula: 100 × (2Na_2_O/0.35 + MgO/0.9 + 2K_2_O/ 0.25 + CaO/0.7). The Chemical Index of Alteration (CIA^104^) was calculated using molar weights of each element as oxides according to the formula: Al_2_O_3_/(Al_2_O_3_ + CaO + Na_2_O + K_2_O) × 100.

### Stable isotope analysis

Pedogenic carbonates were analysed for their stable carbon (δ^13^C) and oxygen (δ^18^O) isotope values from throughout the SGT6 and SGT7 sequences. The rhizomorphs and nodules selected from excavated strata were analogous to both those observed in deflated contexts on the playa surface and in the surrounding dune fields at Singi Talav stand in stark contrast to either powdery calcretes or hardpan calcretes observed elsewhere across the landscape. Up to ten individual carbonates were sampled for each level where available, amalgamated together to form a single sample in each case following Blinkhorn and colleagues^34^.

Every sample of pedogenic carbonate was subjected to an ethanol rinse in order to remove any sediments stuck to the nodule prior to crushing using an agate pestle and mortar. The samples were then dried for 24 h at 40 °C before being placed into borosilicate vials. The vials were flush/filled with helium at 100 ml/min for 10 min. After reaction with 100% phosphoric acid, δ^13^C and δ^18^O measurements were performed on the evolved gases using a Thermo Gas Bench 2 connected to a Thermo Delta V Advantage Mass Spectrometer in the Stable Isotope Laboratory of the Department of Archaeology, Max Planck Institute for the Science of Human History.

δ^13^C and δ^18^O values were compared against those measured for international reference standards (IAEA NBS 18: δ^13^C −5.014 ± 0.032 ‰, δ^18^O −23.2 ± 0.1 ‰, IAEA 603: δ^13^C + 2.46 ± 0.01 ‰, δ^18^O −2.37 ± 0.04 ‰, IAEA CO8: δ^13^C −5.764 ± 0.032 ‰, δ^18^O −22.7 ± 0.2 ‰ and USGS44: δ^13^C =  ~ —42.1 ‰) (n = 3 for each standard). All standards are registered by the International Atomic Energy Agency and were used to calibrate samples using a linear regression methodology. Replicate analysis of in-house MERCK carbonate standards (Merck δ^13^C −41.3 ± 0.1 ‰, δ^18^O −13.4 ± 0.0 ‰) suggests that machine measurement error is *c.* ± 0.1‰ for δ^13^C and ± 0.1‰ for δ^18^O.

### Phytolith analysis

Phytolith analysis was conducted by Dr. Sanjay Eksambekar, Phytolith Research Institute (PRI), Pune, India. Phytolith extraction was undertaken in the laboratory with removal of carbonates and nitrates followed by heavy density separation^105^. Up to 300 phytoliths were observed and counted using an Olympus research microscope with photomicrographs taken under 45X magnification, alongside observations of morphology and preservation. Classifications follow Twiss^106^ and Eksambekar^107^ and were made with reference to the extensive South Asian phytolith database at the PRI.

### Luminescence dating

Samples for luminescence dating were collected by hammering opaque tubes into cleaned sediment faces and were opened and prepared under subdued orange light conditions at the Oxford Luminescence Dating laboratory. Laboratory treatment followed standard procedures (e.g.^34^) to isolate potassium rich feldspar grains for measurement. Elevated temperature post infrared infrared signals measured from very small aliquots (1 mm diameter) of sand-sized grains were used for equivalent dose measurement, and dose rates were derived from radionuclide concentrations determined via inductively coupled plasma mass spectrometry. Final age calculation was undertaken using DRAC^108^. The luminescence dating is discussed in fuller detail in the supplementary information.

## Supplementary Information

Supplementary Information 1.

Supplementary Information 2.

Supplementary Information 3.
