# Supplementary material for: Constraining the chronology and ecology of Late Acheulean and Middle Palaeolithic occupations at the margins of the monsoon
Source: Sci Rep. 2021 Oct 5;11:19665. doi: 10.1038/s41598-021-98897-7 (PMC8492674; doi:10.1038/s41598-021-98897-7)
Supplement: Supplementary file 1 — Supplementary Information 1. [file 41598_2021_98897_MOESM1_ESM.docx]

**Supplementary Information:**

**Constraining the chronology and ecology of Late Acheulean and Middle Palaeolithic occupations at the margins of the monsoon**

James Blinkhorn^1,2^, Hema Achyuthan^3^, Julie Durcan^4^, Patrick Roberts^5^, Jana Ilgner^5^

^1^Pan African Evolution Research Group, Max Planck Institute for the Science of Human History, Jena, Germany

^2^Centre for Quaternary Research, Department of Geography, Royal Holloway, University of London, U. K.

^3^Institute of Ocean Management, Anna University, Chennai, India

^4^School of Geography and the Environment, University of Oxford, U.K.

^5^Department of Archaeology, Max Planck Institute for the Science of Human History, Jena, Germany

Corresponding Author: J. Blinkhorn (blinkhorn@shh.mpg.de)

**SI.1 Previous Investigations at Singi Talav**

Previous research at Singi Talav was undertaken as part of an interdisciplinary project entitled “Early man and his environment in north-west India with special reference to the Luni basin in Rajasthan”, led by Prof V. N. Misra spanning the late 1970’s and 1980’s^1^. Following a series of wide-ranging surveys, a simple division of landforms recognised (from oldest to youngest) the Jayal Formation, a 20-60m thick bed of gravels and boulders likely deposited by a Himalayan river, the Amarpura Formation, composed of heavily calcretised low energy alluvial deposits ranging between 1-2m to 30-40m thick, and the Didwana Formation, comprising more recent aeolian and playa deposition^2^. Rich Acheulean surface sites were identified in the vicinity of Nagaur and Didwana, Rajasthan, including the presence of stratified Acheulean artefacts observed in a calcareous loam quarry in Singi Talav. Excavations were undertaken at the main site (SGT1) between 1981-1985 covering 72m^2^, with an average depth of 1m that exposed 3 discrete artefact bearing horizons, complemented by a 4m^2^ deeper sounding to reveal broader patterns of sedimentation alongside scattered artefacts to a depth of 2m, below which sediments were archaeologically sterile. Two further, shallower test pits were recorded and designated SGT3 and SGT4.

Sediment sequences at Singi Talav can be most simply split between upper aeolian deposits and the calcareous loams on which they rest disconformably. In many instances reporting stratigraphy at the site the interface between aeolian and lacustrine sediments is used as a baseline, rather than the top of a given sediment sequence, which vary more considerably with respect to the scale and preservation of aeolian deposition. This distinction can be readily resolved in terms of sediment texture, the visible presence and density of carbonate nodules within the deposit, and the overall proportion of carbonate within the matrix. A broadly comparable stratigraphic sequence is observed across the site, with the upper reworked aeolian sands (Layers 1-2) sitting disconformably on a calcareous sandy loam, rich in carbonate nodules, (Layers 3-7) which grades into a greyish green, mottled silty loam (Layers 8-11)^3^. The most detailed descriptions of the sediment sequence come from the deep sounding, in which all 11 layers were identified^4^ (Table SI.1).

**Table SI.1:** Description of sediment sequence at Singi Talav exposed in 2x2m deep sounding following extensive excavations at the site during the early 1980’s (modified from^4^).

| **Layer** | **Depth** | **Matrix** | **CaCO_3_** | **Other Clasts/Structure** | **Archaeology** |
| --- | --- | --- | --- | --- | --- |
| 1 | Surface | Yellowish brown sand |  |  | Very rare microliths on the surface |
| 2 | 0-0.18 | Grey silty sand (7.5 YR 7/1), slightly compact | Small round soft CaC03 pellets white in colour, often yellowish on the periphery, Scattered in the matrix  (2 to 5 mm) |  | At the base, accumulation of rolled or wind blasted artifacts |
| 3 | 0.18-0.2 | Uniformly light gray, (10 YR 711) silty clayey sand | Very rich in mainly clast supported CaCO, nodules, indurated and brown in colour, 5 to 15 mm across |  | In the lower part, in-situ artefacts |
| 4 | 0.2-0.5 | Silty clayey sand (7.5 YR 7/1) rich in white powdery CaC03 | Rich in hard brown CaC0_3_, nodules, 5 to  30 mm, even sometimes 50 mm, with Some small soft white  nodules | Coarse alternation of gray layers of silty sand and whitish silt | Rich assemblage with handaxes at the base |
| 5 | 0.5-0.8 | Grayish, sandy silt (2.5 Y 8/1) rather indurated, with some patches of white powdery CaC03 | Rich in small pellety brown CaC03 nodules 5mm on average,  With some bigger ones, brown or white in colour up to 30 mm | Some soft, yellowish pellets of 5mm maximum size (limonite) | Some typical artifacts  fresh or not scattered;  mostly in the upper  horizon |
| 6 | 0.8-1.1 | Gray sandy silt (2.5 Y 8/3) slightly mottled | Rich in relatively hard whitish and very hard brown CaC03  nodules up to 20 mm | Pockets or lenses of soft yellowish  and reddish pellets of  5 mm maximum within a more indurated matrix | Very scarce, but usually  rolled artifacts |
| 7 | 1.1-2 | Gray sandy silt (2.5 Y 8/3) heavily mottled | Rich in hard grayish white nodules, up to  30 mm; no brown CaC03 nodules | Presence of soft yellowish/ reddish pellets and small lenses  (4 cm) of fine red sand in clear bedding planes | A few fresh, well  made flakes at the base, otherwise sterile |
| 8 | 2-2.2 | Grayish-brown compact sandy silt (5 Y 7/2) calcareous | Rich in small (2 to 10mm) hard brown  Clasts of CaC03 pellets  and powder with a few bigger whitish  nodules (25 mm maximum) | Presence of soft yellowish pellets with parallel and obscure bedding planes | Sterile |
| 9 | 2.2-2.7 | Pale olive, mottled silt, loose (5 Y 6/3) | Presence of hard whitish gray nodules  of 20 mm maximum  and of small rounded clasts or CaC03 pellets  (2 to 5 mm) | Thin lenses of fine  reddish sand in obscure  bedding planes | Sterile |
| 10 | 2.7-3.2 | Yellowish brown rather compact silt (10 YR 5/4) | Rich in both small brown (10 mm) and  Also powdery whitish or gray CaC03 nodules  (20 mm) | Dendritic patterns of  Mn/FeO abundant in  the obscure bedding planes | Sterile |
| 11 | 3.2-3.6 | Pale olive and reddish-brown clayey silt (5 Y 6/3) mottled | Poor in hard brownish  white CaC03 pellets with pale brown  nodules of 10 mm diameter | Dendritic patterns of  Mn/FeO present in  the well-marked, obscure bedding  planes through which brackish water seeps | Sterile |

Additional descriptions of the sediment sequence focus on the upper deposits which yielded archaeological assemblages (Tables SI.2-6). Broad comparability in the description of sediments can be identified although some variability in the depth of individual units is present, and particularly at SGT3, which we synthesise in Table SI.7. Of particular relevance here, Unit 3, appearing as the top unit of the calcareous loams, ranges from 0.02 to 0.95m in depth, though in three sections this is constrained to 0.3-0.55m. More muted variability is observed in other key archaeological horizons including Unit 4 varies from 0.25-0.4m, and Unit 5 from 0.3-0.5m.

**Table SI.2**: Description of sediment sequence from Singi Talav^2^

| Unit | Depth | Description |
| --- | --- | --- |
| 1 | 0-0.08 | Brown silty sand sharp contact to |
| 2 | 0.08-0.25 | Greyish clayey silt sharp contact to |
| 3 | 0.25-0.85 | Greyish Green strongly kankarised clayey loam grades to |
| 4 | 0.85-0.95 | Kankar crust or calcrete grades to |
| 5 | 0.95-1.3 | Greyish green moderately kankarised clayey loam; ferruginous pellets common |

**Table SI.3:** Description of sediment sequence at Singi Talav from initial excavations at Singi Talav^5^

| Unit | Depth | Description |
| --- | --- | --- |
| 1 | 0-0.08 |  |
| 2 | 0.08-0.13 | Brown silty sand and hard grey clay silt with soft kankar nodules |
| 3 | 00.13-0.55 | Light grey silty clay with kankar noduls (5-20mm) |
| 4 | 0.55-0.95 | Grey silty clay with kankar nodules (10-50mm) |
| 5 | 0.95+ | hard greenish grey silty clay with yellow kankar nodules (10-50mm) sometimes making a crust at the top - occurrence of small reddish pellets of iron oxide in the lower part |

**Table SI.4:** Composite description of sediment sequence at Singi Talav from excavations at Singi Talav^6^

| Unit | Depth | Sediment | Carbonates | Structure | Archaeology |
| --- | --- | --- | --- | --- | --- |
| 1 | 0-0.25 | Recent brown-yellow sand |  |  | Rare microliths |
| 2 | 0.25-0.3 | Semi-firm grey silty sand | Small soft white round carbonate nodules 2-5mm |  | Rolled MP at base |
| 3 | 0.3-0.6 | Grey sandy silt | Very rich in carbonate nodules, strongly indurates 5-15mm |  | Industry with rare bifaces |
| 4 | 0.6-0.9 | Sandy silt rich in white powdery calcrete | Rich in indurated carbonates 5-30mm and up to 50mm, with some soft white nodules | Large alternation in grey and white beds rich in calcrete | Rich Acheulean industry |
| 5 | 0.9-1.4 | Firm sandy silt with white powdery calcrete concentrations | Rich in brown carbonate nodules (~5mm) with some larger brown or white nodules 30mm |  | Atypical artefacts in upper part |
| 6 | 1.4-1.8 | Grey-green sandy silt | Rich in indurates white carbonate nodules 20mm, with softer smaller yellow nodules 5mm | Rich pockets of granules in an indurated matrix | Very rare eroded artefacts |
| 7 | 1.8-2.3 | Grey-green sandy silt | Rich in white/grey semi-indurated carbonates 30mm, with yellow or red granules | Fine stratified lenses of red sand | Very rare fresh artefacts |
| 8 | 2.3-2.5 | Indurated grey brown sandy silt | Rich in small hard bridged carbonate nodules 2-10mm with larger white and softer yellow nodules | Bedded | Sterile |
| 9 | 2.5-3 | Grey-green silt | Hard grey carbonate nodules 20mm and small bridged nodules 2-5mm | Bedded with fine lenses of red sand | Sterile |
| 10 | 3-3.5 | Indurated greenish silt | Rich in brown carbonate nodules (10mm) and larger white/grey nodules (20mm) | Bedded with dentritic manganese | Sterile |
| 11 | 3.5-3.9 | Grey-green clayey silt | Rare white-brown semi indurated carbonate nodules 10mm | Bedded with manganese dendrites | Sterile |

**Table SI.5:** Description of Singi Talav quarry section sediment sequence^4^

| Unit | Depth | Description |
| --- | --- | --- |
| 1 | 0-0.17 | Yellowish brown(10YR 5/6), well rounded to subrounded, well sorted silty sand |
| 2 | 0.17-0.25 | Pale grey (2.5 YR 7/2) well rounded to sub-angular, moderately sorted clayey silt |
| 3 | 0.25-0.8 | Greenish gray (5GY 6/1) calcareous loam with well-rounded coarse sand and subangular to angular fine silty sand; poorly to moderately sorted calcitic nodules (5-50mm across), some are soft but often have specks of ferruginous color, mottling is a common feature |
| 4 | 0.8-0.9 | Light grey (5 Y 7/1) well rounded to sub-rounded, moderately sorted silty sand. This layer is rather compact and consists of pelletic brown calcrete nodules (5mm average across) |
| 5 | 0.9-1.3 | Light grey (5 Y 7/1) sub-rounded, moderately sorted, silty sand, slightly calcretized with predominance of soft, as well as rather indurated CaCO3 nodules (20-30mm across); mottling is a common feature |

**Table SI.6**: Description of Singi Talav trench SGT3^6^

| Unit | Depth |  |
| --- | --- | --- |
| 1 | 0-0.25 | Recent brown pale sand |
| 2 | 0.25-0.65 | Compact pale brown silty sand; indurated brown carbonate nodules 100mm max but dispersed |
| 3 | 0.65-1.6 | Grey sandy silt; rich in indurated rounded brown carbonate nodules 5-15mm, with some bridging |
| 4 | 1.6-1.85 | Grey sandy silt with diffuse carbonate powder; rich in little carbonate nodules, white, rounded |

**Table SI.7:** Synthesis of strata depths previously reported from Singi Talav.

| Unit | Misra et al. 1982 | Gaillard et al. 1985 | Raghaven et al. 1991 (Quarry) | Raghavan et al. 1991 (Deep Sounding) | Gaillard 1993 Synthesis | Gaillard 1993 SGT3 |
| --- | --- | --- | --- | --- | --- | --- |
| 2 | 0.17 | 0.07 | 0.08 | 0.18 | 0.05 | 0.4 |
| 3 | 0.6 | 0.42 | 0.55 | 0.02 | 0.3 | 0.95 |
| 4 | 0.1 | 0.4 | 0.1 | 0.3 | 0.3 | 0.25 |
| 5 | 0.35 |  | 0.4 | 0.3 | 0.5 |  |
| 6 |  |  |  | 0.2 | 0.4 |  |
| 7 |  |  |  | 0.9 | 0.6 |  |
| 8 |  |  |  | 0.2 | 0.2 |  |
| 9 |  |  |  | 0.5 | 0.5 |  |
| 10 |  |  |  | 0.5 | 0.5 |  |
| 11 |  |  |  | 0.4 | 0.3 |  |

More detailed description of the sediments includes the results of sieve grain size analysis, with individual samples processed for each layer^6^, and the proportion of CaCO_3_ present identified by the insoluble mineral residue method, with multiple samples processed spanning Layers 2-5^4^. We used Gradistat^7^ to characterise the grain size analysis data^6^ to facilitate comparative analyses (Table SI.8). Cogley and Aikmen^8^ have cautioned that the insoluble mineral residue method consistently overestimates actual proportions of carbonate present by up to 20% in carbonate rich sediments, such as those present at Singi Talav. The original % CaCO_3_ values are reported in Table SI.9 alongside adjusted values following ^8^.

**Table SI.8**: New summary of grain size characteristics from Singi Talav, produced from previously reported results.

| Level | Type | Name | Skewness | Kurtosis | Mean (ɸ) | Sorting | Skewness | Kurtosis |
| --- | --- | --- | --- | --- | --- | --- | --- | --- |
| **2** | Unimodal, Poorly Sorted | Very Fine Silty Very Fine Sand | Very Fine Skewed | Extremely Leptokurtic | 3.7 | 1.7 | 1.4 | 5.4 |
| **3** | Bimodal, Poorly Sorted | Very Coarse Silty Very Fine Sand | Very Fine Skewed | Very Leptokurtic | 3.9 | 1.6 | 0.7 | 3.8 |
| **4** | Polymodal, Very Poorly Sorted | Coarse Silty Very Fine Sand | Very Fine Skewed | Leptokurtic | 4.6 | 2.4 | 0.8 | 2.7 |
| **5** | Polymodal, Very Poorly Sorted | Coarse Silty Very Fine Sand | Very Fine Skewed | Mesokurtic | 4.7 | 2.5 | 0.6 | 2.4 |
| **6** | Polymodal, Very Poorly Sorted | Very Fine Sandy Coarse Silt | Very Fine Skewed | Mesokurtic | 5.5 | 2.4 | 0.5 | 2.3 |
| **7** | Polymodal, Very Poorly Sorted | Very Fine Sandy Coarse Silt | Fine Skewed | Platykurtic | 5.6 | 2.6 | 0.1 | 2.0 |
| **8** | Polymodal, Very Poorly Sorted | Very Fine Sandy Coarse Silt | Fine Skewed | Platykurtic | 5.6 | 2.5 | 0.0 | 2.4 |
| **9** | Polymodal, Very Poorly Sorted | Very Fine Sandy Coarse Silt | Fine Skewed | Leptokurtic | 5.5 | 2.4 | 0.2 | 2.4 |
| **10** | Trimodal, Very Poorly Sorted | Very Fine Sandy Mud | Very Fine Skewed | Very Platykurtic | 5.8 | 2. 5 | 0.4 | 1.9 |
| **11** | Bimodal, Poorly Sorted | Fine Sandy Coarse Silt | Symmetrical | Very Leptokurtic | 5.3 | 1.9 | 0.2 | 3.6 |

**Table SI.9**: Proportion of CaCO_3_ in sediment samples from Singi Talav^4^, resulting from insoluble mineral residue method alongside adjusted values following^8^.

| Unit | Depth (m) | % Carbonate | Adjusted % Carbonate |
| --- | --- | --- | --- |
| 2 | 0.16 | 12.7 | 10.1 |
| 2 | 0.19 | 13.6 | 10.9 |
| 2 | 0.22 | 17.9 | 14.3 |
| 2 | 0.25 | 21.3 | 17 |
| 3 | 0.28 | 54.4 | 43.5 |
| 3 | 0.32 | 55.6 | 44.5 |
| 3 | 0.37 | 67.3 | 53.9 |
| 3 | 0.42 | 56.4 | 45.1 |
| 3 | 0.47 | 66.6 | 53.3 |
| 3 | 0.52 | 60.9 | 48.8 |
| 3 | 0.57 | 62.1 | 49.7 |
| 3 | 0.62 | 62.5 | 50 |
| 3 | 0.67 | 66.1 | 52.9 |
| 3 | 0.77 | 62.1 | 49.7 |
| 4 | 0.87 | 68 | 54.4 |
| 5 | 0.92 | 68.3 | 54.7 |
| 5 | 1.07 | 61.8 | 49.4 |
| 5 | 1.25 | 55.3 | 44.2 |
| 5 | 1.35 | 56 | 44.8 |

Weathered artefacts reported as microliths and Middle Palaeolithic artefacts were recovered from the surface (Layer 1) and upper deposits (Layer 2) at the site respectively, with two dense assemblages recovered from Layers 3 (n=401) and 4 (n=891), and more diffuse artefacts recovered from Layer 5 (n=173), all three of which were reported as Acheulean industries and predominately appear in fresh condition^6,9^; see SI.3 for further details). Further artefacts were recovered from Layers 6 and 7 but are not documented. Varied quartzites available in the nearby Balia Hills (ca. 3km) form the mainstay of raw materials used, with a few artefacts likely deriving from alternate materials available in cobble beds up to 20km away. In addition to stone tools, six quartz crystals recovered from the excavations appear to be manuports, and have been argued to reflect selection for their aesthetic appeal^10,11^. The presence of small, unweathered or rolled gastropod shells and opercula supports suggestions that primary activities at the site were conducted on the shore of an ancient playa, and were rapidly buried^3^.

**SI.2 Previous assessment of the chronology of Singi Talav**

No previous direct dating has been undertaken at Singi Talav. Preliminary exploration of Quaternary deposits in the region led Misra and colleagues^2^ to suggest an Early Late Pleistocene to Middle Pleistocene age to the Amarpura formation, to which the sediment sequence at Singi Talav had been attributed. The initial dating of the nearby 16R Dune sequence appeared consistent with this suggestion, with Lower Palaeolithic artefacts reported dating from 390ka, and Middle Palaeolithic artefacts dating from 144ka^12^. Further refinement of the chronology of the 16R Dune sequence suggests U/Th measurement presented maximum ages, and, in combination with luminescence dating, suggested a younger chronology at the site^13,14^. Artefact collections attributed to the Lower Palaeolithic are constrained between dates of 109±20ka and 187±43ka, although the absence of clearly diagnostic Acheulean tool types prohibit clear characterisation of this technology^15^. The larger Middle Palaeolithic occupation at the site is now constrained by ages of 40±3ka and 80±10ka, though likely closer to the latter^15^.

Kailath and colleagues^16^ undertook the first experimental use of Electron Spin Resonance (ESR) dating in India, studying carbonated from a range of settings in the Thar Deserts included a sample from Amarpura Quarry, located approximately 2km from Singi Talav. The methodological limitations of this study are clearly caveated by these researchers, and particular problems are noted for the sample studied from Amarpura, such as mismatching palaeodosimetry signals. The results are clearly stated with caution: “It is suggested that the ages be taken as notional age estimates only” (^16^:381). The notional age estimate from Amarpura is reported as 797ka, with the researchers noting this was not consistent with the anticipated age range based on geomorphology and sample morphology, or to comparable geomorphologies with similar sample morphologies which returned ages of 328-53ka^16^. Kailath and colleagues^16^ do not report the stratigraphic sequence of provenance of the sample that was analysed, though Gaillard and colleagues^17^ indicate that the sample was recovered from 1m below the top of carbonate rich deposit. The stratigraphy from the quarry reported by Misra and colleagues^2^ in 1982 is summarised in Table SI.10, however this may not reflect the sequence that was sampled in 2000.

**Table SI.10:** Typical stratigraphy at Amarpura Quarry reported in 1982, following Misra and colleagues^2^

| Layer | Depth | Description |
| --- | --- | --- |
| 1 | 0-1.1 | Brownish, weakly kankarised aeolian sand, sharp contact to |
| 2 | 1.1-3.6 | Greyish green, strongly kankarised concretionary loam, sharp contact to |
| 3 | 3.6-3.85 | Calcrete, grades to |
| 4 | 3.85-4.75 | Pinkish brown, strongly kankarised sand loam, sharp contact to |
| 5 | 4.75-4.95 | Calcrete, grades to |
| 6 | 4.95-5.95 | Brown calcareous sandy loam, grades to |
| 7 | 5.95-6.05 | Greyish silty clay with soft carbonate segregates, grades to |
| 8 | 6.05-7 | Greyish, moderately kankarised clayey silt with soft ferruginous pellets |

Based on the shared presence of calcretised loamy sediments at Singi Talav and Amarpura (both within the Didwana basin) and Acheulean artefacts, notably four handaxes, the age estimate from Amarpura of 797ka has been suggested to provide a minimum age estimate for the Acheulean occupations of Singi Talav^3,17–19^. This attribution has been critiqued by Chauhan^20^, who highlights the absence of detailed provenance of the dating sample, lack of details regarding the stratigraphic profile sampled, demonstration of any clear stratigraphic continuity between the two sites, and emphasises the significant distance between them, questioning the reliability of correlations between the sites whilst reiterating the experimental nature of results presented by Kailath and colleagues^16^. This is compounded by our field assessment of the topography of the landscape, supported by analysis of an ALOS DEM that suggests that the land surface at Amarpura sits approximately 10m above that of Singi Talav (Figure SI.1). Given the experimental nature of the date for the Amarpura deposits, the absence of demonstrated continuity between them and Singi Talav, and the starkly different topographic position of these locations, it is not tenable to attribute the minimum age of 797ka to the Singi Talav archaeological horizons. Notably, however, the chronometric age estimates we present for deposits at Singi Talav (SI5) are consistent with other samples analysed by Kailath and colleagues^16^ from comparable sheet washed aggraded plain deposits that were not subject to similar experimental issues and range between 328-53ka.


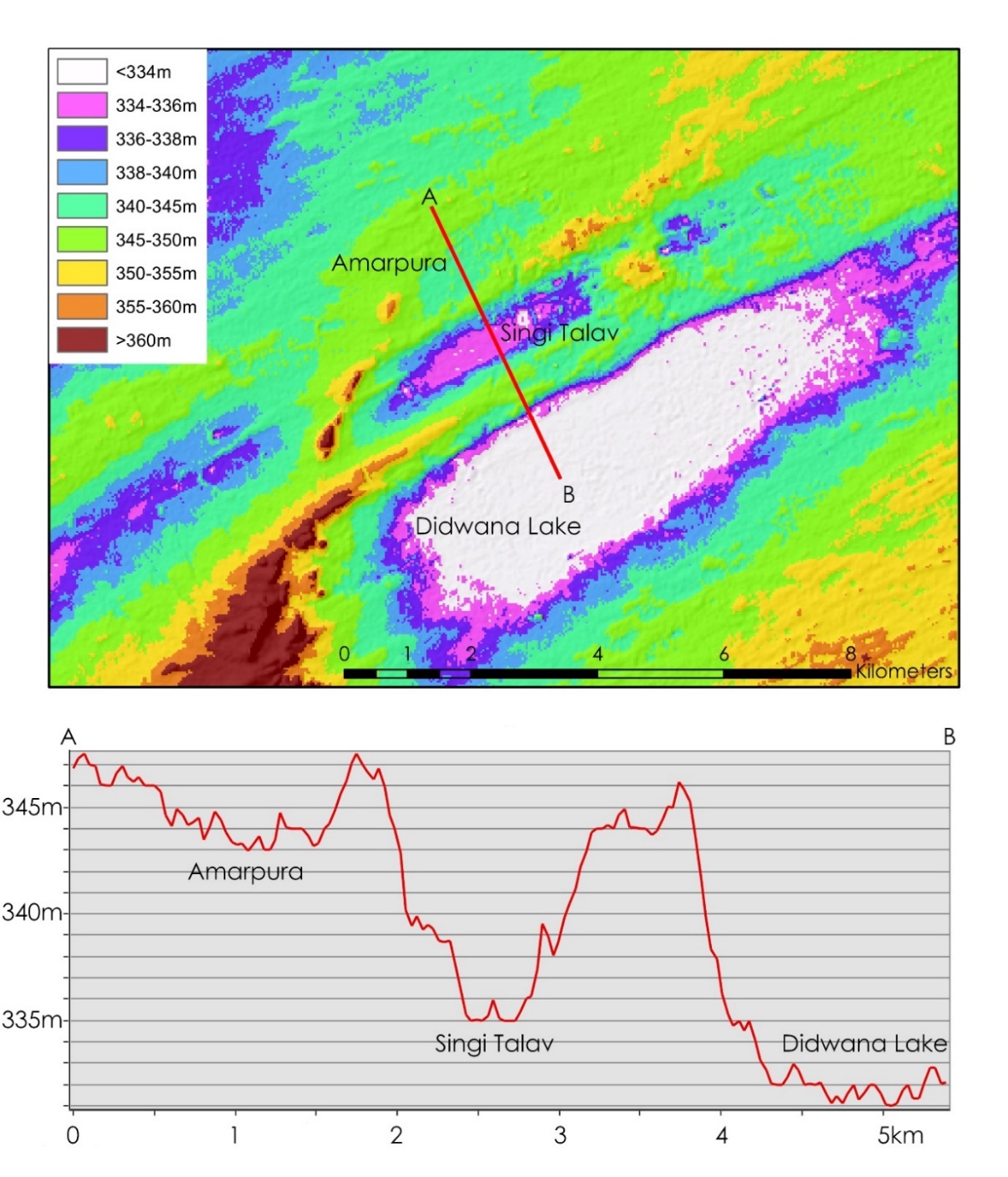


**Figure SI.1:** (top) ALOS DEM (Data: ALOS [JAXA]) illustrating the location of Amarpura, Singi Talav and Didwana Lake and the position of cross section AB; (bottom) topographic profile along cross section AB, highlighting the locations of Amarpura, Singi Talav, and Didwana Lake, illustrating an 8-10m difference in the surface height between Amarpura and Singi Talav, produced using ArcMAP 10.5 (www.esri.com).

**SI.3 New Investigations at Singi Talav**

We undertook new investigations at Singi Talav, continuing the numbering of sections examined from previous work at the site. We examined three sites (SGT5-7) and here report the results from SGT6 and SGT7. Detailed results following laboratory analyses are appended as SI.5

**SGT6**

SGT6 was located within close proximity to the edge of the abandoned quarry at Singi Talav, where erosion of the quarry edge clearly exposed artefacts eroding from stratified calcareous deposits. A 0.8m deep section was excavated in a 1x1m trench, with ingress of water (relating to industrial activity at the site, rather than the natural water table) prohibiting deeper excavation. Through the combination of field descriptions of sediment colour, texture, and macroscopic components (e.g. size, density and induration of carbonate nodules), laboratory analyses of fine sediments, and statistical analyses of the results, we resolve between five discrete sediment units at SGT6 (summarised in Table SI1.11), which closely correspond to previous descriptions of the upper five units reported from the main excavation sites at Singi Talav (see SI.1). Mean grain sizes from each layer at SGT6 (Table SI.12) closely match those from the original excavations (Table SI.8), with comparable trends observed in changes in sorting, skewness and kurtosis though the sequence. The increasing proportion of CaCO_3_ within fine sediments between Layers 2-4 are consistent between sites, including the peak values observed at the top of Layer 5. Similarly, the distribution of lithic artefacts closely parallels patterns reported from previous excavations at the site.

**Table SI11:** Summary description of sediment layers from SGT6 sequence, which are directly comparable with the layers identified from previous excavations.

| Layer | Depth (m) | Description |
| --- | --- | --- |
| 1 | 0-0.15 | A unimodal, pale grey very coarse silty fine sand, with sparse, soft, small carbonate nodules (<5mm), with sparse lithic artefacts |
| 2 | 0.15-0.45 | A uni- to trimodal pale grey very coarse silty fine sand preserving common, small, firm carbonate nodules (~5mm) with sparse lithic artefacts, concentrated in a horizon at 0.35m |
| 3 | 0.45-0.55 | A polymodal pale grey coarse silty fine sand with frequent, larger and more compacted carbonate nodules (5-15mm) with rare lithic artefacts |
| 4 | 0.55-0.75 | A polymodal pale grey fine sand to medium silt with dense, large, compact carbonate nodules (~10-15mm) with rare lithic artefacts |
| 5 | 0.75+ | A bimodal pinkish grey fine sandy medium silt with dense, fusing carbonate nodules (5-15mm) and fine ferric nodules (<5mm) and powdery calcretes with a single lithic artefact identified |

**Table SI12:** Summary results of LPSA and LOI analyses from SGT6 reported by layer, illustrating close comparability with trends identified from the previously excavated sequences (Table SI8).

| Layer | Mean (ɸ) | Sorting | Skewness | Kurtosis | %CaCO_3_ (LOI) |
| --- | --- | --- | --- | --- | --- |
| 1 | 3.38 | 1.89 | 0.87 | 3.58 | 9.70 |
| 2 | 3.65 | 2.07 | 0.84 | 3.11 | 24.16 |
| 3 | 3.86 | 2.24 | 0.37 | 2.43 | 42.56 |
| 4 | 4.42 | 2.13 | 0.22 | 2.28 | 63.26 |
| 5 | 4.87 | 2.22 | -0.23 | 2.30 | 71.39 |

**SGT7**

SGT7 was located approximately 400m from the original excavation site, with the section available for study cleaned by heavy plant machinery as part of ongoing industrial use of the site. This enabled us to capitalise upon visibility of a significantly deeper sediment sequence within the constraints of modern activity at Singi Talav. Through the combination of field descriptions of sediment colour, texture, and macroscopic components (e.g. size, density and induration of carbonate nodules), laboratory analyses of fine sediments, and statistical analyses of the results, we resolve between eight discrete sediment units at SGT7 (summarised in Tables SI.13). Broad similarities can be identified with the uppermost five units identified in previous excavations, including a notable change to heavily calcretised sediments (Layer 3 and below), the frequency and appearance of carbonate nodules, the decreasing trend in mean sediment size, sorting, skewness and kurtosis of fine sediments, and the increase in proportion of CaCO_3_ present in the fine sediment fraction followed by a limited decline in the upper part of Layer 5 (Table SI.14). Below Layer 5, a divergence is apparent in terms of fine sediment grain size characteristics, with results from SGT7 indicating higher proportions of very fine sands to coarser silts than the deep sounding from the original excavations. While these differences may have resulted in the alternate methods used in grain size analyses, it is also likely that different parts of the site may have received different scales of sediment inputs from aeolian activity, which may also account for these differences. Comparable changes in the sediment sequence are, however, observed in terms of levels of sediment compaction, changes in colour, the presence of ferric and manganese nodules, and in the size and extent of bridging observed for carbonate nodules present. Despite some divergence in grain size characteristics at the site, based on multiple shared characteristics, we correlate the SGT7 sequence with the upper eight layers from the earlier excavations, with divergences in the lower three levels acknowledged by attributing them to Layers 6*, 7* and 8*. In additional to laboratory results presented in SI.5, we present counts of phytoliths from each sample from SGT7 in Table SI.15.

**Table SI13:** Summary description of sediment layers from SGT7 sequence, which are directly comparable with the layers identified from previous excavations.

| Layer | Depth | Description |
| --- | --- | --- |
| 1 | 0-0.55 | A unimodal, mid grey, very coarse silty fine sand, with rare fine carbonate nodules (<5mm) |
| 2 | 0.55-0.65 | A bimodal pale brownish grey very coarse fine silty sand with small, soft carbonate nodules (5mm) |
| 3 | 0.65-1.00 | A bi- to trimodal mid brownish grey fine sandy silt with common, compact carbonate nodules (5-15mm) |
| 4 | 1.00-1.15 | A polymodal pale brownish grey very fine sandy coarse silt with common, compact carbonate nodules (5-15mm) with rare ferric mottles |
| 5 | 1.15-1.6 | A trimodal pale brownish grey fine sand and coarse silt with concentrated powdery calcrete and frequent, compact carbonate nodules (5-15mm) |
| 6* | 1.6-1.85 | An upward fining, polymodal, mid orangish brown very fine sandy coarse silt with dense, firm, bridged carbonate nodules (5-15mm) |
| 7* | 1.85-2.05 | An upward coarsening, hard, poly-modal, mid reddish brown very coarse silt to fine sand with common indurated and bridged carbonate nodules (5-15mm) |
| 8* | 2.05-2.35 | An indurated, polymodal, dark reddish brown very coarse silty very fine sand with frequent indurated and bridged carbonate nodules (5-20mm) and fine ferric/manganese nodules |

**Table SI14:** Summary results of LPSA and LOI analyses from SGT7 reported by layer, illustrating close comparability with trends identified from the previously excavated sequences (Table SI8).

| Layer | Mean (ɸ) | Sorting | Skewness | Kurtosis | %CaCO3 (LOI) |
| --- | --- | --- | --- | --- | --- |
| 1 | 3.71 | 1.91 | 0.92 | 3.58 | 7.33 |
| 2 | 4.01 | 2.19 | 0.40 | 2.39 | 27.43 |
| 3 | 4.68 | 2.17 | 0.12 | 2.26 | 45.15 |
| 4 | 4.62 | 2.06 | 0.16 | 2.21 | 61.67 |
| 5 | 4.04 | 2.33 | 0.19 | 2.28 | 49.36 |
| 6* | 4.51 | 2.24 | 0.04 | 2.55 | 55.03 |
| 7* | 4.09 | 2.21 | 0.27 | 2.57 | 43.12 |
| 8* | 3.82 | 2.20 | 0.39 | 2.77 | 34.60 |

**Table SI15:** Counts of phytolith types from SGT7 listed by depth

| Depth | Panicoid | Chloridoid | Festucoid | Elongate | Trichome | Bulliform | Woody elements |
| --- | --- | --- | --- | --- | --- | --- | --- |
| 0-5cm | 22 | 50 | 41 | 52 | 81 | 28 | 26 |
| 10-15cm | 27 | 48 | 23 | 89 | 55 | 33 | 25 |
| 20-25cm | 12 | 33 | 18 | 52 | 73 | 68 | 44 |
| 30-35cm | 23 | 19 | 21 | 61 | 67 | 86 | 23 |
| 40-45cm | 15 | 12 | 18 | 58 | 73 | 98 | 26 |
| 50-55cm | 58 | 23 | 33 | 53 | 57 | 55 | 21 |
| 60-65cm | 43 | 31 | 38 | 69 | 54 | 33 | 32 |
| 70-75cm | 12 | 43 | 49 | 31 | 81 | 59 | 25 |
| 80-85cm | 17 | 34 | 72 | 81 | 23 | 39 | 34 |
| 90-95cm | 32 | 18 | 34 | 77 | 67 | 48 | 24 |
| 100-105cm | 11 | 31 | 46 | 23 | 73 | 84 | 32 |
| 110-115cm | 18 | 34 | 73 | 39 | 64 | 32 | 40 |
| 120-125cm | 41 | 18 | 12 | 53 | 48 | 54 | 74 |
| 130-135cm | 37 | 16 | 23 | 49 | 51 | 53 | 71 |
| 140-145cm | 87 | 41 | 18 | 48 | 45 | 37 | 24 |
| 150-155cm | 68 | 39 | 24 | 36 | 54 | 41 | 38 |
| 160-165cm | 54 | 33 | 28 | 59 | 57 | 25 | 44 |
| 170-175cm | 47 | 19 | 28 | 76 | 48 | 66 | 16 |
| 180-185cm | 38 | 22 | 31 | 79 | 46 | 74 | 10 |
| 190-195cm | 53 | 32 | 19 | 31 | 74 | 77 | 14 |
| 200-205cm | 44 | 15 | 21 | 66 | 56 | 59 | 39 |
| 210-215cm | 69 | 58 | 46 | 42 | 27 | 39 | 19 |
| 220-225cm | 52 | 34 | 64 | 81 | 23 | 30 | 16 |
| 230-235cm | 43 | 28 | 53 | 61 | 37 | 40 | 38 |

**SI.4 Stone tool technology at Singi Talav**

Detailed studies of the stone tool collections from the 1981-1985 excavations at Singi Talav were conducted by Gaillard^6^, and recently reviewed by Gaillard and Rajaguru^9^, primarily describing the assemblages within the major categories of flakes, debris, retouched flake tools and small/medium core tools, large core tools, and large cutting tools. The reported artefact inventories from Layers 3, 4 and 5 are summarised in Tables SI.16-17. More limited description of the Layer 2 assemblage has been reported, with no detailed information available for other archaeological horizons. The initial excavation report details the Layer 2 assemblage comprising 196 artefacts, including a handaxe, two choppers, three chopping tools and a scraper, alongside flakes and debris. These artefacts are noted as distinctly smaller than the underlying assemblages and is described as Middle Palaeolithic. The three assemblages best documented from Singi Talav (Layers 3-5) are described as Acheulean, and predominantly made on varying quartzites and quartzes, with rare pieces of schist. These raw materials appear to have been sourced locally, from the Balia Hills ca. 3km from the site, although up to 5% of artefacts are thought to have been produced on river cobbles that are likely sourced from the Jayal Gravel Ridge ca. 20km from the site. Common classes of artefacts present include retouched flake tools, such as scrapers, and large cutting tools, such as handaxes. Between 5-8% of the assemblages are classified as small, medium or large core tools, typically showing bifacial or multifacial working and interpreted as possessing working edges. While a large proportion of the flakes are described as the result of large cutting tool reduction sequences, the presence of alternate debitage schemes to produce discrete flake products are indicated^6^. Renewed examination of these collections are required in the light of detailed technical descriptions of alternate debitage approaches, such as Levallois^21,22^ and discoidal reduction^23^, to better illuminate the presence and diversity debitage reduction strategies present at the site. This is particularly pertinent as the presence and diversity of alternate core reduction strategies sits at the crux of differences between Late Acheulean and Middle Palaeolithic technologies, both in the Thar Desert^24^, and elsewhere across the region^25–27^.

**Table SI.16**: Proportions of alternate artefact classes recovered from 1981-1985 excavations at Singi Talav in Layers 3, 4 and 5, following Gaillard^6^ and Gaillard and Rajaguru^9^.

| **Class** | **3** | **4** | **5** |
| --- | --- | --- | --- |
| Flakes | 42% | 40% | 34% |
| Debris | 43% | 46% | 56% |
| Retouched Flake Tools | 6% | 7% | 4% |
| Small/Medium Core Tools | 2% | 2% | 2% |
| Large Core Tools | 6% | 3% | 4% |
| Large Cutting Tools | 1% | 3% | - |
| Total (n) | 401 | 891 | 181 |

**Table SI.17**: Counts of alternate artefact types recovered from 1981-1985 excavations in Layers 3, 4, and 5 at Singi Talav, following Gaillard^6^ and Gaillard and Rajaguru^9^.

| **Class** | **Type** | **3** | **4** | **5** |
| --- | --- | --- | --- | --- |
| Debitage | Flakes | 167 | 357 | 61 |
|  | Debris | 171 | 408 | 101 |
| Retouched Flake Tools | Beak | 1 | 7 |  |
|  | Burin |  | 3 | 1 |
|  | Composite Tool | 8 | 15 |  |
|  | Convergent Tool/Point | 5 | 5 |  |
|  | Denticulate | | 3 |  |
|  | Denticulate Scraper | 3 | 6 |  |
|  | End Scraper | 4 | 7 |  |
|  | Notch | 2 | 8 | 1 |
|  | RT Point | |  | 1 |
|  | RT Tool |  |  | 2 |
|  | Side Scraper | 2 | 7 | 2 |
| Medium Tools/Small Core Tools | Core/Core Tools | |  | 4 |
|  | Micro-Chopper | 2 |  |  |
|  | Micro-Chopping Tool | 2 | 7 |  |
|  | Micro-core (Multi-Facial) | 1 | 3 |  |
|  | Micro-Discoid (Bifacial) | | 4 |  |
|  | Micro-Polyhedron (Multifacial) | 2 | 3 |  |
|  | Micro-Spheroid with edge (Bifacial) | 1 |  |  |
|  | Micro-Spheroid without edge (Bifacial) | | 1 |  |
| Larger Core Tools | Atypical Chopper | 2 | 1 |  |
|  | Atypical Chopping Tool | 4 | 1 |  |
|  | Chopping Tool | 2 | 5 |  |
|  | Convergent Chopper | 1 |  |  |
|  | Core (Multi-Facial) | 5 | 1 |  |
|  | Discoid (Bifacial) | 1 | 3 |  |
|  | Double Chopper/Chopping Tool | 1 | 4 |  |
|  | Hammerstone | 1 | 1 |  |
|  | Polyhedron (Multi-Facial) | 1 | 2 |  |
|  | Single Removal (Core Tool) | 3 | 2 |  |
|  | Spheroid with edge (Bifacial) | 5 | 3 |  |
| Large Cutting Tools | Cleaver |  | 3 |  |
|  | Handaxe (Bifacial) | 3 | 18 |  |
|  | LCT |  |  | 8 |
|  | Pick |  | 1 |  |
|  | Proto-Handaxe | 1 | 1 |  |
|  | Uniface |  | 1 |  |
| Total | | 401 | 891 | 181 |

Investigation at SGT6 resulted in the recovery of a small collection of artefacts (n=56; Table SI.18) from buried contexts, which parallel the recovery of stone tool assemblages from previous excavations, most notably the artefact densities/depths as initially reported by Misra and colleagues^2^.

**Table SI.18:** Artefacts recovered from investigation at SGT6.

| **Artefact Type** | **Layer 1** | **Layer 2** | **Layer 3** | **Layer 4** | **Layer 5** |
| --- | --- | --- | --- | --- | --- |
| Flake | 7 | 9 | 3 | 3 | 1 |
| Flaked Piece | 12 | 4 | 1 | 5 |  |
| Multi-Platform Core | 1 | 2 | 1 |  |  |
| Core on Flake | 3 | 1 |  |  |  |
| Discoidal Core | 1 |  |  | 1 |  |
| Retouched Flake |  |  | 1 |  |  |

**SI.5 Luminescence Dating**

*Equivalent dose measurement and calculation*

Samples for luminescence dating were collected by hammering opaque tubes into cleaned sediments faces, which were subsequently transferred to the Oxford Luminescence Dating laboratory where they were opened under subdued orange light conditions. Prior to laboratory treatment, the light exposed sediment at the ends of the sample tube was removed and used for dose rate determination. Preparation of sediment for measurement of luminescence signals followed standard treatment protocols, where sediments were treated with hydrochloric acid and hydrogen peroxide to remove carbonates and organic matter respectively, prior to sediment sieving and heavy liquid density separation to isolate the potassium feldspar mineral component (<2.58 g.cm^-3^). Sediments were not etched with hydrofluoric acid to avoid the possibility of anisotropic removal of the grain surface (e.g.^28^). Very small aliquots (1 mm diameter) of purified potassium feldspar (90-150 μm grain size range) were adhered onto 9.7 mm aluminium discs for measurement.

Post infrared infrared (pIRIR) stimulated luminescence measurements were made using a Risø TL/OSL DA-15, fitted with 870 nm infrared LEDs for stimulation. Samples were irradiated with a ^90^Sr/^90^Y beta source with a dose rate of approximately 4 Gy/min. Blue feldspar luminescence signals were detected through a bialkali photo multiplier tube fitted with BG-39 and Corning 7-59 filters. Following pre-heat and dose recovery tests, the pIRIR signal measured at 225°C (pIRIR_225_) was selected for measurement of the equivalent doses (D_e_), using the pIRIR protocol shown in Table SI.19. D_e_s were calculated from the pIRIR_225_ signal measured during the first 2 s of stimulation, with the mean background over the last 10 s of stimulation subtracted. Dose response curves were fitted with an exponential plus linear function, selected on the basis of the lowest reduced chi-squared value. Luminescence signals were screened using a suite of standard rejection criteria, and signals were only accepted for age calculation if; i) the test dose response was at least three sigma greater than background, 2) the recycling ratio was within 10% of unity (including associated uncertainties), and 3) recuperation was less than 5%. In addition, signals considered to be in saturation (where D_e_ exceeds 2D0, including uncertainties) were also excluded from final D_e_ calculation. De distributions showed low levels of overdispersion (Table SI.20), and on this basis, the central age model (CAM) of Galbraith and colleagues^29^ was used for final D_e_ calculation.

**Table SI.19:** The pIRIR_225_ SAR protocol used in this study, following Smedley and colleagues^30^.

| **Step** | **Treatment** |
| --- | --- |
| 1 | Dose |
| 2 | Preheat at 250°C for 60 s |
| 3 | IRSL at 50°C for 100 s |
| 4 | pIRIR at 225°C for 100 s |
| 5 | Test dose (ca. 110 Gy) |
| 6 | Preheat at 250°C for 60 s |
| 7 | IRSL at 50°C for 100 s |
| 8 | pIRIR at 225°C for 100 s |
| 9 | IRSL at 290°C for 100 s |

*Testing the use of the pIRIR_225_ luminescence signal*

Residual dose and dose recovery tests were carried out on sample ST6-1 to assess the suitability of the pIRIR_225_ luminescence signal and measurement protocol. Prior to all measurements, very small aliquots of sample were bleached in daylight conditions for 10 days to remove the bleachable pIRIR_225_ signal. To test the bleachability of the pIRIR_225_ signal, a residual-dose test was carried out to establish how much signal remained after the daylight bleach. The measured residual D_e_ remaining after the daylight bleach was 11.2 ± 2.06 Gy (n = 6 discs) (Figure SI2). A dose recovery test (n = 12) was then carried out using a laboratory dose of ca. 175 Gy. This resulted in a mean residual-subtracted dose-recovery ratio of 0.90 ± 0.04, demonstrating the suitability of the selected protocol and signal for dating.


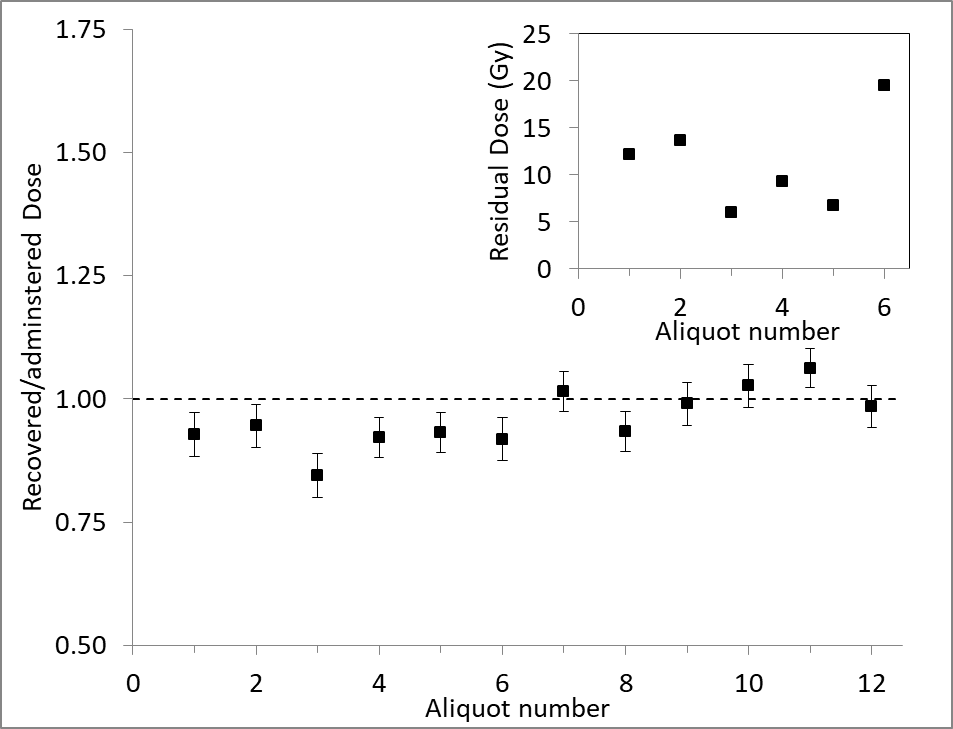


**Figure SI.2:** Individual dose recovery test results from 12 very small aliquots of sample ST6-1 (recovered dose 165 Gy), and inset, the measured residual dose after a 10-day daylight exposure.

It has been reported that some pIRIR luminescence signals are not thermally stable, which requires the measurement of and potential adjustment for anomalous fading. A fading test was performed on 3 very small aliquots of each sample to determine whether the pIRIR_225_ signal from the Singi Talav sediments were athermally stable. To determine the fading rate (g-value), a laboratory dose approximate to the natural luminescence signal was measured promptly, and after 1, 10 and 100 hour delays, using the method of Huntley and Lamothe^31^, and g-values were normalised to two days^32^. Following the approach of Smedley and colleagues^30^, g-values were calculated for each individual aliquot (Figure SI3) before the weighted mean and standard error of the whole dataset was calculated. For the pIRIR_225_ signal, average g-values of 1.0 ± 0.5 %/decade were calculated. The pIRIR_225_ value is consistent with other published values (e.g. ^33,34^), as well as values published from the region (e.g. ^35,36^). This value is also consistent with g-values calculated for quartz signals (e.g. ^37,38^), which are not routinely fading-corrected. Given this, pIRIR_225_ signals in this study have not been corrected (e.g. ^30^).


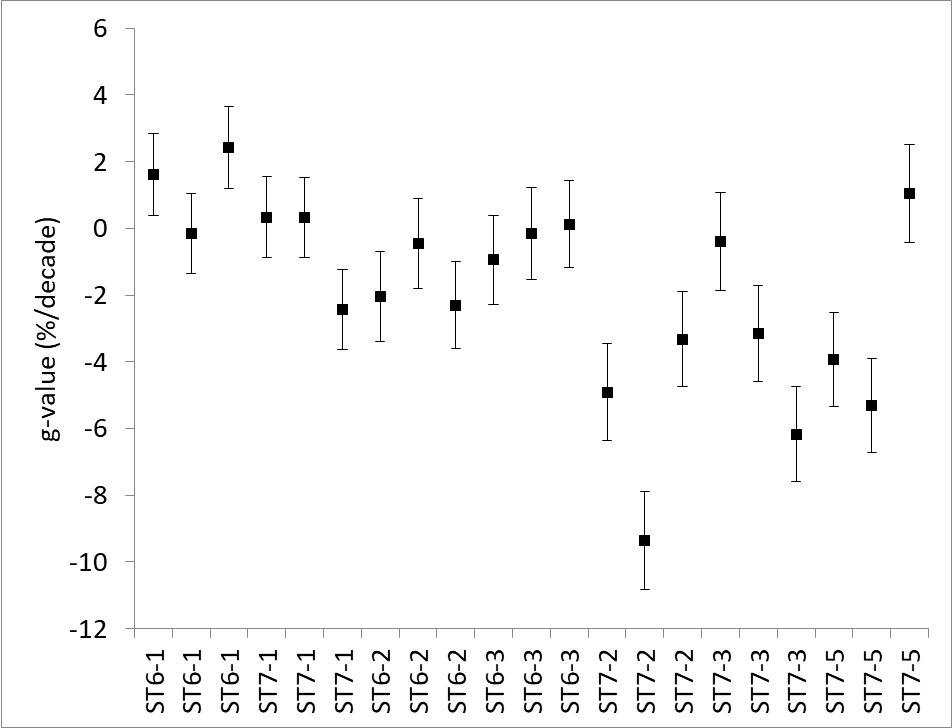


**Figure SI.3:** Individual g-values (normalised to two days), calculated from 3 very small aliquots of each sample. Negative g-values are viewed as indicative of negligible fading. Laboratory doses of 165 Gy (samples ST6/1, ST7/1), 385 Gy (ST6/2, ST6/3) and 550 Gy (ST7/2, ST7/3, ST7/5) were selected for use, to be representative of the natural dose and to fall within signal saturation limits. As detailed in the main text, after dosing, luminescence measurements were made immediately, and after 1, 10, and 100-hour delays.

*Equivalent dose distributions*

The Singi Talav samples yield bright pIRIR_225_ luminescence signals (e.g. Figure SI4), and all measured signals satisfied the rejection criteria relating to signal behaviour (recycling ratio, recuperation, sensitivity) and are considered suitable for dating. In addition to these rejection criteria, signal saturation was considered. There is no standardised approach for dealing with saturated signals ^39^, although the dose level characteristic of the dose response curve, or the D_0_ parameter, has been proposed in the past^40^ as a criterion for identifying such signals, with 2 D_0_ suggested as a prudent upper limit for D_e_. From the 168 measured signals, 2D_0_ varies between 235 and 830 Gy, with an average of 560 ± 119 Gy. In order to avoid erroneous age calculations from less precise, potentially saturated D_e_s, signals where D_e_>2D_0_ (including uncertainties) were not included in final D_e_ calculations, and the majority of signals satisfied this criterion. However, a proportion of D_e_s from samples ST7-2 and ST7-3 (Table SI.4) did not satisfy this, and were excluded from final D_e_ calculation. For these two samples, we provide a finite age (based on 7 and 11 aliquots respectively; Table SI.4). Disregarding the 2D0 saturation criteria did not result in extensively older ages, and we also provide these in Table SI.15.

Dose distributions, including the excluded saturated signals are shown in Figure SI5. Overdispersion, or variability in dose distribution beyond that expected from intrinsic luminescence properties alone, values are provided in Table SI.15 and range between 0 and 21%. The lower overdispersion values tend to occur for dose distributions containing individual D_e_s with lower precision, and these are typically higher D_e_s (e.g. samples ST7-2, -3, -5), interpolating from the dose response curve as it approaches the asymptote (e.g. ^41^). Nonetheless, the observed range of overdispersion for these samples is in line with other reported values from luminescence dating of lake shorelines (e.g. ^42^). With the low levels of overdispersion observed, the central age model^29^ was used for final D_e_ calculation.

**Table SI.20:** Equivalent dose summary. For samples ST7-2, 7-3, and 7-5, some signals did not satisfy the 2D_0_ saturation criterion (see text). For comparison, we show the calculated ages with the criterion applied, and not (in italics).

| Sample | Grain size (μm) | # Accepted (measured) aliquots | Overdispersion (%) | CAM D_e_ (Gy) | Dose Rate (Gy.ka^-1^) | Age (ka) |
| --- | --- | --- | --- | --- | --- | --- |
| ST6-1 | 90-150 | 24 (24) | 18.8±0.6 | 188.96±7.42 | 2.90±0.24 | 65.14±5.28 |
| ST6-2 | 90-150 | 24 (24) | 8.4±0.4 | 406.16±8.79 | 2.30±0.22 | 176.67±16.83 |
| ST6-3 | 90-150 | 22 (24) | 8.2±0.6 | 485.6±12.26 | 1.96±0.21 | 248.14±26.75 |
| ST7-1 | 90-150 | 24 (24) | 20.9±0.7 | 285.65±13.16 | 3.35±0.25 | 85.27±6.26 |
| ST7-2 | 90-150 | 7 (24)  *24 (24)* | 2.8±2.9  4.6±0.9 | 754.34±26.14  *799.26±19.65* | 3.18±0.24 | 237.44±18.16  *251.58±19.24* |
| ST7-3 | 90-150 | 11 (24)  *24 (24)* | 3.6±2.1  *4.5±1.3* | 823.83±27.99  *867±24.57* | 3.17±0.25 | 260.21±20.14  *274.16±21.22* |
| ST7-5 | 90-150 | 20 (24)  *24 (24)* | 4.3±1.3  4.3±1.1 | 683.39±19.77  *687.60±19.21* | 3.69±0.26 | 185.2±13.05  *186.34±13.13* |


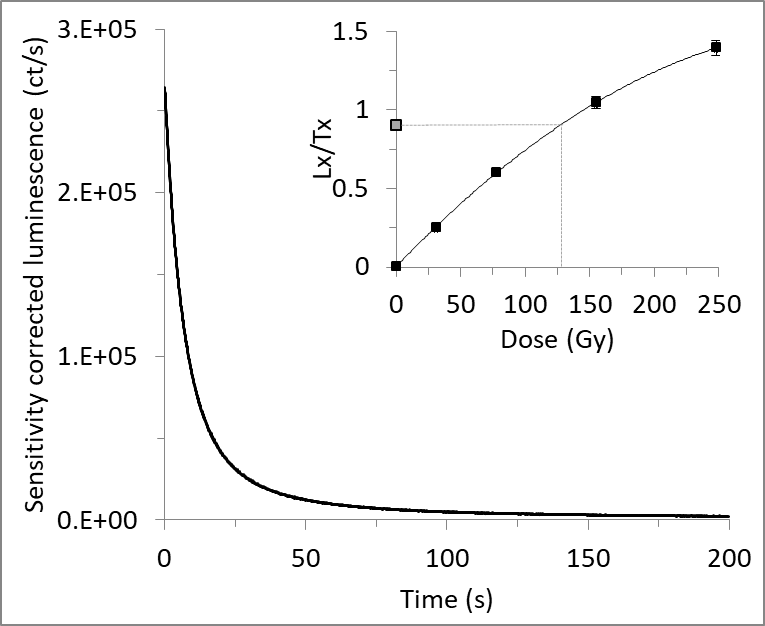


**Figure SI.4:** Example pIRIR_225_ signal and dose response curve (inset) from a very small aliquot of sample ST6-1, with a D_e_ of 128.4 Gy.

| ST6-1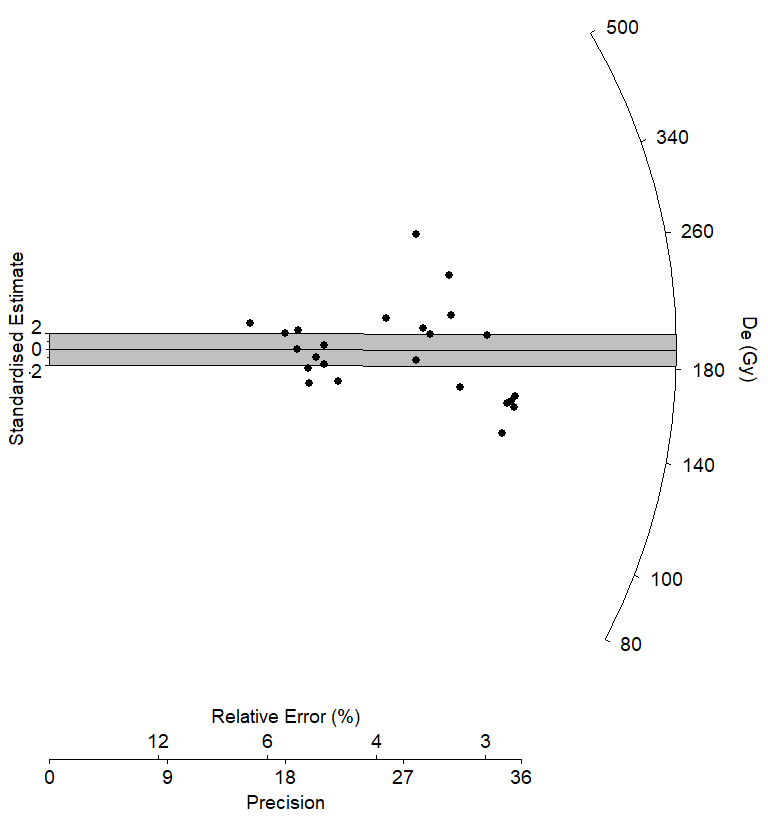 | ST6-2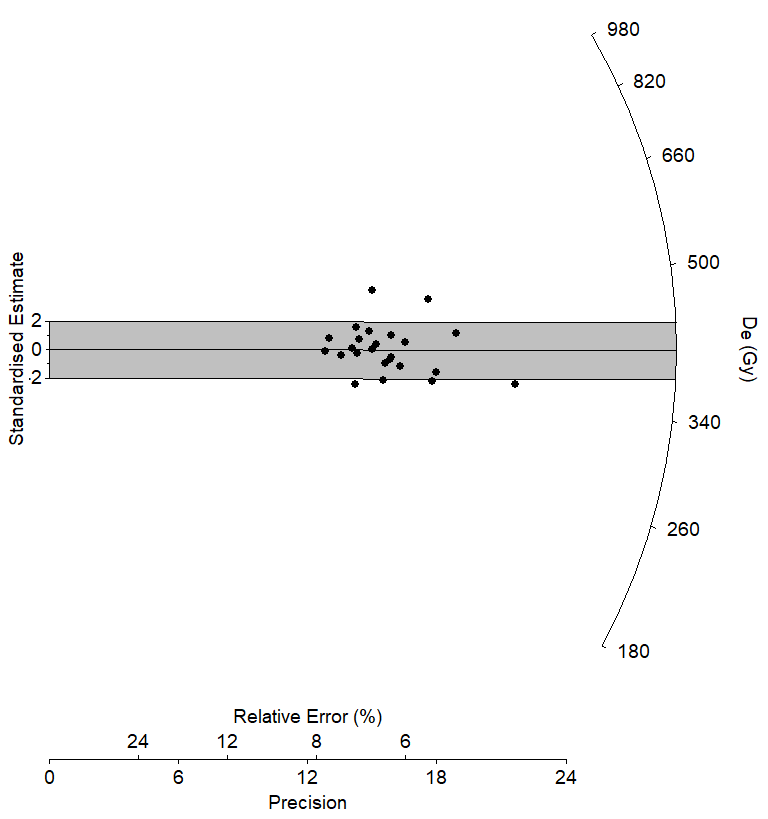 |
| --- | --- |
| ST6-3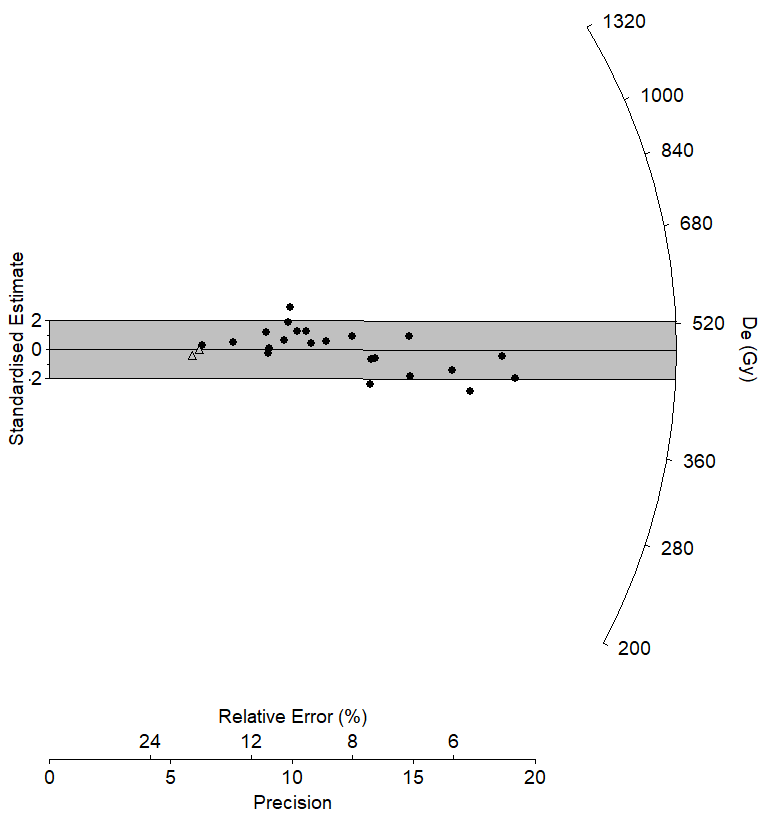 | ST7-1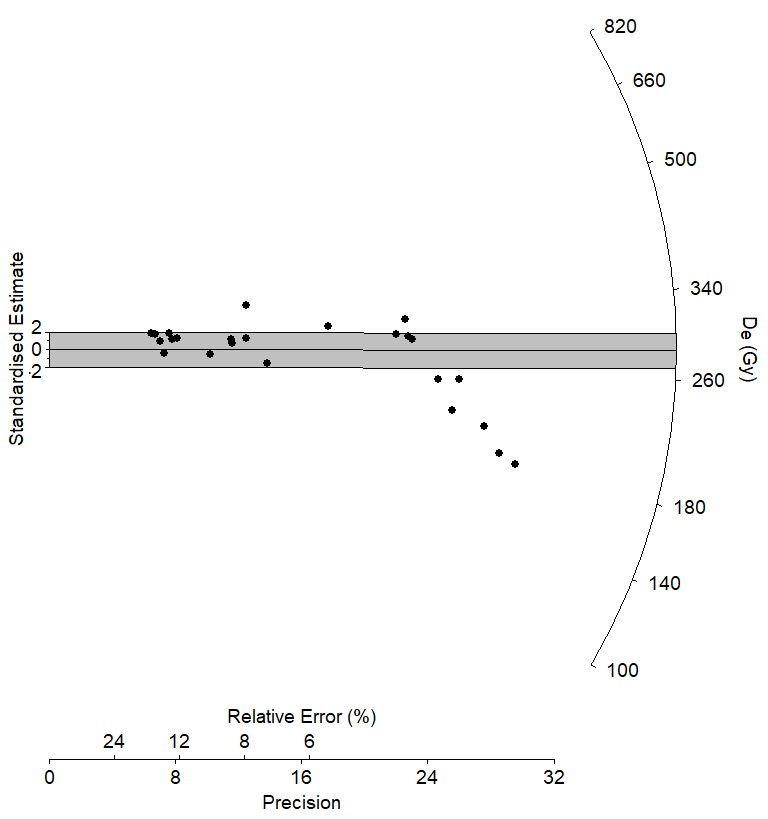 |
| ST7-2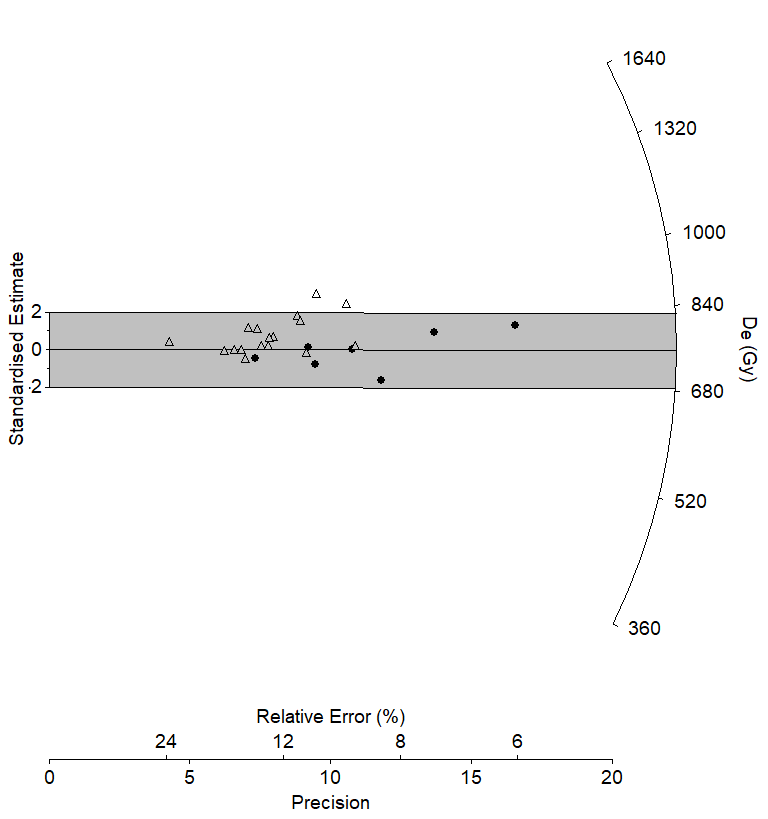 | ST7-3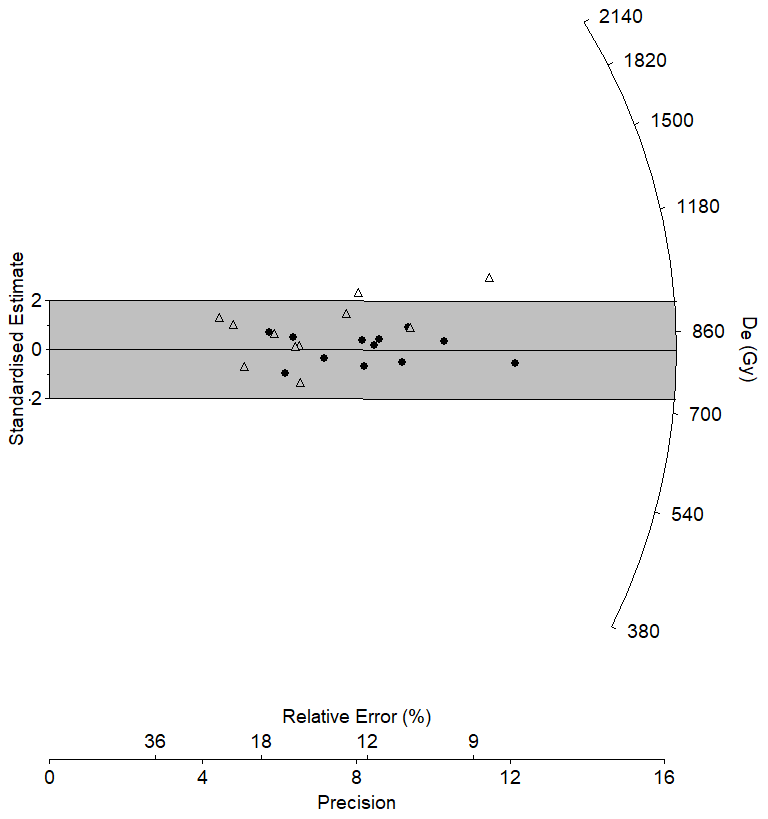 |
| ST7-5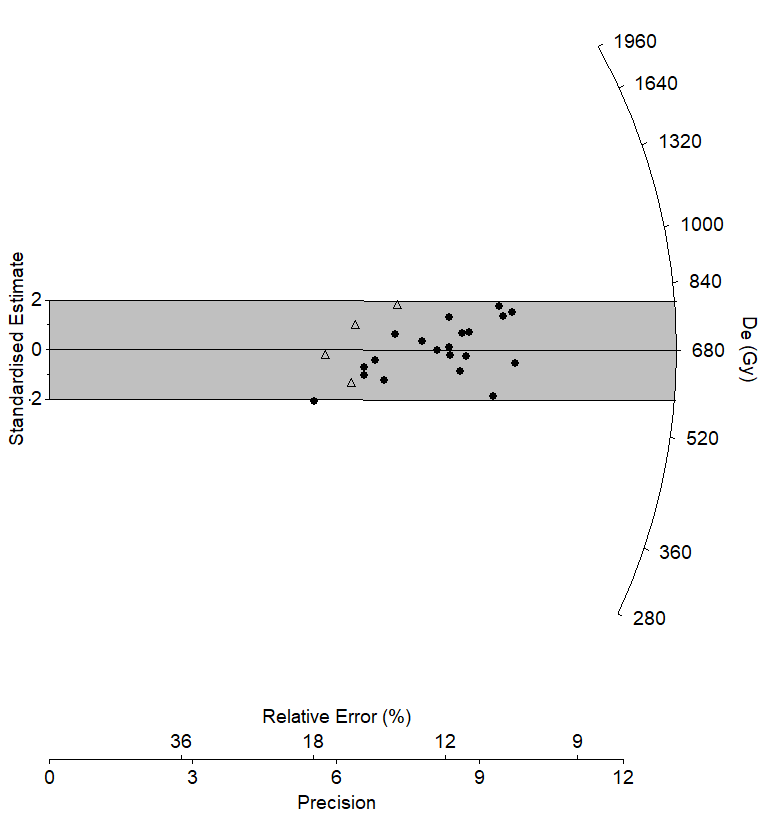 |  |

**Figure SI.5:** Radial plots showing D_e_ distributions. The dark grey band is centred on the sample D_e_ (black line), with all points falling within the grey band consistent with this D_e_ (within 2σ). Closed symbols indicate individual D_e_ values which satisfy all rejection criteria. Open symbols show D_e_s which satisfy the rejection criteria but were excluded from final D_e_ calculation due to signal saturation (D_e_ >2D_0_ including uncertainties).

*Dose rate measurement and calculation*

Environmental dose rates were calculated using the DRAC software of Durcan and colleagues^43^. Radionuclide concentrations, measured from oven-dried, homogenised sediment using inductively coupled plasma mass spectrometry (ICP-MS), were converted to dose rates using the feldspar conversion factors of Guerin and colleagues^44^. Infinite-matrix dose rates were adjusted for attenuation by grain size and chemical etching using the factors of Brennan and colleagues ^45^, Guerin and colleagues ^44^, and Bell^46^. A moisture content of 5 ± 2 % was assumed, following the rationale of Neudorf and colleagues^35^ and Durcan and colleagues^47^ for samples taken from fluvial contexts where precipitation is delivered seasonally via the Indian Monsoon System. Alpha dose rates were corrected for alpha efficiency using an a-value of 0.11 ± 0.03^48^, and an internal potassium content of 10 ± 2 % was used to calculate the internal beta dose rate^49^. Calculation of the cosmic dose rate was calculated based on the geographic location and sampling depth of each sampling, following Prescott and Hutton^50^. Individual dose rates were combined, with uncertainties propagated in quadrature. Dose rate data are summarised in Table SI.21.

**Table SI.21:** Dose rate summary.

| Sample | U (ppm) | Th (ppm) | K (%) | Dose Rate (Gy.ka^-1^) | | | | | |
| --- | --- | --- | --- | --- | --- | --- | --- | --- | --- |
|  |  |  |  | Alpha | Internal Beta | External Beta | Gamma | Cosmic | Total |
| ST6-1 | 1.32±0.13 | 8.00±0.80 | 1.35±0.13 | 0.13±0.05 | 0.43±0.20 | 1.29±0.10 | 0.82±0.05 | 0.23±0.02 | 2.90±0.24 |
| ST6-2 | 1.13±0.11 | 4.96±0.50 | 1.04±0.10 | 0.09±0.03 | 0.43±0.20 | 0.99±0.08 | 0.59±0.04 | 0.21±0.02 | 2.30±0.22 |
| ST6-3 | 1.35±0.14 | 3.90±0.39 | 0.72±0.07 | 0.09±0.03 | 0.43±0.20 | 0.76±0.06 | 0.49±0.03 | 0.19±0.02 | 1.96±0.21 |
| ST7-1 | 4.34±0.43 | 6.94±0.69 | 1.12±0.11 | 0.22±0.08 | 0.43±0.20 | 1.47±0.11 | 1.04±0.07 | 0.19±0.02 | 3.35±0.25 |
| ST7-2 | 3.62±0.36 | 5.57±0.56 | 1.26±0.13 | 0.18±0.06 | 0.43±0.20 | 1.46±0.11 | 0.93±0.06 | 0.18±0.02 | 3.18±0.24 |
| ST7-3 | 4.94±0.49 | 5.99±0.60 | 0.86±0.09 | 0.23±0.08 | 0.43±0.20 | 1.34±0.10 | 1.00±0.07 | 0.17±0.02 | 3.17±0.25 |
| ST7-5 | 2.56±0.26 | 11.72±1.17* | 1.61±0.16 | 0.21±0.08 | 0.43±0.20 | 1.71±0.13 | 1.18±0.08 | 0.16±0.02 | 3.69±0.26 |

^*^Further discussion of the Th concentration for sample ST7-5 can be found in-text.

Dose rate determination for luminescence dating assumes that ionisation of the sample has remained constant over the burial period, and that the decay chains of Uranium and Thorium are in equilibrium. Dose rates calculated in this study were made on the basis of this assumption, although sedimentary changes involving water can result in disequilibrium^51–53^. In this suite of samples, we record values for Uranium between 1.13 and 4.94 ppm, Th between 3.9 and 8.00 ppm (with the 11.7 ppm value for sample ST7-5 discussed further below), and K between 0.7 and 1.6%. These values are consistent with values recorded at other sites regionally, where for example Blinkhorn and colleagues^54^ measure ranges of 2.3-4.3 ppm, 4.1-7.5 ppm, and 0.1-0.7% of U, Th, and K respectively at Nal Quarry site in the central Thar, derived from XRF and ICP-MS measurements. Our measured values are also in line with other luminescence studies in the Thar region^47,55,56^. Of note is the uncharacteristically high concentration of Thorium for sample ST7-5 (11.7 ppm in contrast with 6ppm for the overlying sample ST7-3) (Table SI.21). This may be indicative of post depositional Th inputs to the sediment, and the potential for disequilibrium, which would result in an inflated dose rate and age underestimation. Whilst the sampling strategy sought to reduce the potential for dosimetric complications, by sampling away from sedimentary boundaries, from carbonate material, and evidence for water fluctuation (e.g.^53^), given the stratigraphic age inversion, we hypothesise that the calculated dose rate may not reflect the long term dose rate for sample ST7-5. Given this inflated value for Th, and the resulting age inversion (Table SI.20), we suggest that the calculated age likely underestimates the true burial age of this sample.

When dating potassium feldspar grains, the dose rate derived from potassium sources both internal and external to the grains must be taken into account during dose rate determination, resulting in a relatively high contribution from this radionuclide. In this study, on average K accounts for 50% of the total dose rate, with the cosmic dose rate comprising on average 7.5% of the total, resulting in ~60% of the dose rate derived from these two sources which are not affected by disequilibria (individual dose rates from all sources for all samples are provided in the DRAC output file SI7). Olley and colleagues^57^ suggest that an error of up to 8% may be introduced where substantial (e.g. 50%) disequilibrium exists. Should this be the case, this would not affect the findings of this study. However, given that the total dose rate is dominated by inputs from potassium and the cosmic dose rates, that radionuclide concentrations measured are consistent with regional values, and that the luminescence ages are in stratigraphic order (excluding ST7-5, as discussed), we are confident that disequilibrium issues are not overly impactful in this study.

**References Cited:**

1. Misra, V. Geoarchaeology of the Thar desert, northwest India. *Mem. Soc. INDIA* 210–230 (1995).

2. Misra, V. N., Rajaguru, S. N., Raju, D. R., Raghavan, H. & Gaillard, C. Acheulian Occupation and Evolving Landscape Around Didwana in the Thar Desert, India. *Man Environ.* **VI**, 72–86 (1982).

3. Rajaguru, S. N., Deo, S. G. & Gaillard, C. Pleistocene Geoarchaeology of Thar Desert. **53**, 63–76 (2014).

4. Raghavan, H., Gaillard, C. & Rajaguru, S. N. Genesis of Calcretes from the Calc-pan Site of Singi Talav Near A Micromorphological Approach. *Geoarchaeology* **6**, 151–168 (1991).

5. Gaillard, C., Misra, V. N., Rajaguru, S. N., Raju, D. R. & Raghavan, H. Acheulian occupation at Singi-Talav, in the Thar desert : a preliminary report on 1981 excavation. *Bull. Deccan Coll. Res. Inst.* **44**, 141–152 (1985).

6. Gaillard, C. Contribution à la connaissance du paléolithique inférieur-moyen en Inde. (Université de Provence, 1993).

7. Blott, S. J. & Pye, K. Technical Communication Gradistat : a Grain Size Distribution and Statistics Package for the Analysis of Unconsolidated Sediments. *Earth Surf. Process. Landforms* **26**, 1237–1248 (2001).

8. Cogley, J. G. & Aikman, M. The insoluble residue test for abundance of carbonate. *Earth Surf. Process. Landforms* **22**, 1053–1059 (1997).

9. Gaillard, C. & Rajaguru, S. N. Revisiting the Acheulian site of Singi Talav at Didwana (Rajasthan) 35 years. in *Rethinking the Past: A Tribute to Professor V. N. Misra* (ed. Deo, S. G.) 25–39 (Indian Society for Prehistoric and Quaternary Studies, 2017). doi:10.7765/9780719098451.00013.

10. d’Errico, F., Gaillard, C. & Misra, V. N. Collection of non-utilitarian objects by Homo erectus in India. in *Hominidae: Proceedings of the 2nd International Congress of Human Paleontology* (ed. Giacobini, G.) 237–239 (Jaca Book, 1989).

11. Moncel, M.H., L. Chiotti, L., Gaillard, C., Onoratini, G. & Pleurdeau, D. Non-utilitarian objects in the Palaeolithic: Emergence of the sense of precious? *Archaeol. Ethnol. Anthropol. Eurasia* **40**, 24–40 (2012).

12. Raghavan, H., Rajaguru, S. & Misra, V. Radiometric dating of a Quaternary dune section, Didwana, Rajasthan. *Man Environ.* **13**, 19–22 (1989).

13. Achyuthan, H., Quade, J., Roe, L. & Placzek, C. Stable isotopic composition of pedogenic carbonates from the eastern margin of the Thar Desert, Rajasthan, India. *Quat. Int.* **162**, 50–60 (2007).

14. Singhvi, A. K. *et al.* A ~200 ka record of climatic change and dune activity in the Thar Desert, India. *Quat. Sci. Rev.* **29**, 3095–3105 (2010).

15. Blinkhorn, J. A new synthesis of evidence for the Upper Pleistocene occupation of 16R Dune and its southern Asian context. *Quat. Int.* **300**, 282–291 (2013).

16. Kailath, A. J. *et al.* Electron spin resonance characterization of calcretes from Thar desert for dating applications. *Radiat. Meas.* **32**, 371–383 (2000).

17. Gaillard, C., Mishra, S., Singh, M., Deo, S. & Abbas, R. Reply to: “Comment on ‘lower and early Middle Pleistocene Acheulian in the Indian sub-continent’” by P. Chauhan. *Quat. Int.* **223**–**224**, 260–264 (2010).

18. Gaillard, C. Les premiers peuplements d’Asie du Sud : vestiges culturels. *Comptes Rendus Palevol* **5**, 359–369 (2006).

19. Gaillard, C., Mishra, S., Singh, M., Deo, S. & Abbas, R. Lower and Early Middle Pleistocene Acheulian in the Indian sub-continent. *Quat. Int.* **223**–**224**, 234–241 (2010).

20. Chauhan, P. R. Comment on [`]Lower and Early Middle Pleistocene Acheulian in the Indian sub-continent’ by Gaillard et al. (2009) (Quaternary International). *Quat. Int.* **223**–**224**, 248–259 (2010).

21. Boeda, E. *Le concept Levallois, Variabilité des méthodes.* (CNRS, 1994).

22. Van Peer, P. *Levallois Reduction Sequence*. (Prehistory Press, 1992).

23. *Discoid Lithic Technology*. (BAR Publishing, 2003).

24. Blinkhorn, J. The gateway to the oriental zone: Environmental change and palaeolithic behaviour in the Thar Desert. *Quat. Int.* **596**, 79–92 (2021).

25. Haslam, M. *et al.* Indian lithic technology at the time of the 74kyr BP Toba super-eruption: New evidence from Kurnool District, Andhra Pradesh. (2011).

26. Clarkson, C. *et al.* Human occupation of northern India spans the Toba super-eruption ~74,000 years ago. *Nat. Commun.* **11**, (2020).

27. Akhilesh, K. *et al.* Early Middle Palaeolithic culture in India around 385-172 ka reframes out of Africa models. *Nature* **554**, 97–101 (2018).

28. Duller, G. A. T. Luminescence chronology of raised marine terraces, south-west North Island, New Zealand. 147 (1992).

29. Galbraith, R. F., Roberts, R. G., Laslett, G. M., Yoshida, H. & Olley, J. M. Optical dating of single and multiple grains of quartz from jinmium rock shelter, northern australia: part i, experimental design and statistical models*. *Archaeometry* **2**, 339–364 (1999).

30. Smedley, R. K., Glasser, N. F. & Duller, G. A. T. Luminescence dating of glacial advances at Lago Buenos Aires (~46 °S), Patagonia. *Quat. Sci. Rev.* **134**, 59–73 (2016).

31. Huntley, D. J. & Lamothe, M. Ubiquity of anomalous fading in K-feldspars and the measurement and correction for it in optical dating. *Can. J. Earth Sci.* **38**, 1093–1106 (2001).

32. Auclair, M., Lamothe, M. & Huot, S. Measurement of anomalous fading for feldspar IRSL using SAR. *Radiat. Meas.* **37**, 487–492 (2003).

33. Trauerstein, M., Lowick, S. E., Preusser, F. & Schlunegger, F. Small aliquot and single grain IRSL and post-IR IRSL dating of fluvial and alluvial sediments from the Pativilca valley, Peru. *Quat. Geochronol.* **22**, 163–174 (2014).

34. Roberts, H. M. Testing Post-IR IRSL protocols for minimising fading in feldspars, using Alaskan loess with independent chronological control. *Radiat. Meas.* **47**, 716–724 (2012).

35. Neudorf, C. M., Roberts, R. G. & Jacobs, Z. Assessing the time of final deposition of Youngest Toba Tuff deposits in the Middle Son Valley, northern India. *Palaeogeogr. Palaeoclimatol. Palaeoecol.* **399**, 127–139 (2014).

36. Blinkhorn, J. *et al.* The fi rst directly dated evidence for Palaeolithic occupation on the Indian coast at Sandhav , Kachchh. *Quat. Sci. Rev.* **224**, 105975 (2019).

37. Buylaert, J. P. *et al.* A robust feldspar luminescence dating method for Middle and Late Pleistocene sediments. *Boreas* **41**, 435–451 (2012).

38. Thiel, C. *et al.* Luminescence dating of the Stratzing loess profile (Austria) - Testing the potential of an elevated temperature post-IR IRSL protocol. *Quat. Int.* **234**, 23–31 (2011).

39. Stewart, M. *et al.* Human footprints provide snapshot of last interglacial ecology in the Arabian interior. *Sci. Adv.* **6**, eaba8940 (2020).

40. Wintle, A. G. & Murray, A. S. A review of quartz optically stimulated luminescence characteristics and their relevance in single-aliquot regeneration dating protocols. *Radiat. Meas.* **41**, 369–391 (2006).

41. Murray, A. S., Wintle, A. G., Wallinga, J., Horowitz, Y. S. & Oster, L. Dose estimation using quartz OSL in the non-linear region of the growth curve. *Radiat. Prot. Dosimetry* **101**, 371–374 (2002).

42. Thomas, D. S. G., Bailey, R., Shaw, P. A., Durcan, J. A. & Singarayer, J. S. Late Quaternary highstands at Lake Chilwa, Malawi: Frequency, timing and possible forcing mechanisms in the last 44 ka. *Quat. Sci. Rev.* **28**, 526–539 (2009).

43. Durcan, J., King, E. G. & Duller, G. A. T. DRAC : Dose Rate and Age Calculator for trapped charge dating DRAC : Dose Rate and Age Calculator for trapped charge dating. *Quat. Geochronol.* **28**, 54–61 (2015).

44. Guérin, G., Mercier, N., Nathan, R., Adamiec, G. & Lefrais, Y. On the use of the infinite matrix assumption and associated concepts: A critical review. *Radiat. Meas.* **47**, 778–785 (2012).

45. Brennan, B. J., Lyons, R. G. & Phillips, S. W. Attenuation of alpha particle track dose for spherical grains. *Int. J. Radiat. Appl. Instrumentation. Part D. Nucl. Tracks Radiat. Meas.* **18**, 249–253 (1991).

46. Bell, W. T. Attenuation factors for the absorbed radiation dose in quartz inclusions for thermoluminescence dating. *Anc. TL* **8**, 1–12 (1979).

47. Durcan, J. A. *et al.* Holocene landscape dynamics in the Ghaggar-Hakra palaeochannel region at the northern edge of the Thar Desert, northwest India. *Quat. Int.* **501**, 317–327 (2019).

48. Balescu, S. & Lamothe, M. Thermoluminescence dating of the holsteinian marine formation of Herzeele, northern France. *J. Quat. Sci.* **8**, 117–124 (1993).

49. Smedley, R. K., Duller, G. A. T., Pearce, N. J. G. & Roberts, H. M. Determining the K-content of single-grains of feldspar for luminescence dating. *Radiat. Meas.* **47**, 790–796 (2012).

50. Prescott, J. R. & Hutton, J. T. Cosmic ray contributions to dose rates for luminescence and ESR dating: Large depths and long-term time variations. *Radiat. Meas.* **23**, 497–500 (1994).

51. Ivanovich, M. & Harmon, R. S. *Uranium-series disequilibrium: Applications to Earth, Marine, and Environmental Sciences*. (Clarendon Press, 1992).

52. Abdualhadi, S. A., Mauz, B., Joss, S. D. T. & Nolan, P. J. Detecting and quantifying uranium-series disequilibrium in natural samples for dosimetric dating applications. *Radiat. Meas.* **114**, 25–31 (2018).

53. Degering, D. & Degering, A. Change is the only constant - time-dependent dose rates in luminescence dating. *Quat. Geochronol.* **58**, 101074 (2020).

54. Blinkhorn, J., Achyuthan, H., Jaiswal, M. & Singh, A. K. The first dated evidence for Middle-Late Pleistocene fluvial activity in the central Thar Desert. *Quat. Sci. Rev.* **250**, (2020).

55. Srivastava, A., Durcan, J. A. & Thomas, D. S. G. Analysis of late Quaternary linear dune development in the Thar Desert, India. *Geomorphology* **344**, 90–98 (2019).

56. Srivastava, A., Thomas, D. S. G. & Durcan, J. A. Holocene Dune Activity in the Thar Desert, India. *Earth Surf. Process. Landforms* **44**, 1407–1418 (2019).

57. Olley, J. M., Murray, A. & Roberts, R. G. The effects of disequilibria in the uranium and thorium decay chains on burial dose rates in fluvial sediments. *Quat. Sci. Rev.* **15**, 751–760 (1996).
